# Globally elevated excitation–inhibition ratio in children with autism spectrum disorder and below-average intelligence

**DOI:** 10.1186/s13229-022-00498-2

**Published:** 2022-05-12

**Authors:** Viktoriya O. Manyukhina, Andrey O. Prokofyev, Ilia A. Galuta, Dzerassa E. Goiaeva, Tatiana S. Obukhova, Justin F. Schneiderman, Dmitrii I. Altukhov, Tatiana A. Stroganova, Elena V. Orekhova

**Affiliations:** 1 Center for Neurocognitive Research (MEG Center), Moscow State University of Psychology and Education, Moscow, Russian Federation; 2 Department of Psychology, National Research University Higher School of Economics, Moscow, Russian Federation; 3 MedTech West and the Institute of Neuroscience and Physiology, Sahlgrenska Academy, The University of Gothenburg, Gothenburg, Sweden

**Keywords:** Autism spectrum disorders (ASDs), Intelligence, Magnetoencephalography, Power spectrum, 1/f power law, Excitation–inhibition balance, Biomarkers

## Abstract

**Background:**

Altered neuronal excitation–inhibition (E–I) balance is strongly implicated in ASD. However, it is not known whether the direction and degree of changes in the E–I ratio in individuals with ASD correlates with intellectual disability often associated with this developmental disorder. The spectral slope of the aperiodic 1/f activity reflects the E–I balance at the scale of large neuronal populations and may uncover its putative alternations in individuals with ASD with and without intellectual disability.

**Methods:**

Herein, we used magnetoencephalography (MEG) to test whether the 1/f slope would differentiate ASD children with average and below–average (< 85) IQ. MEG was recorded at rest with eyes open/closed in 49 boys with ASD aged 6–15 years with IQ ranging from 54 to 128, and in 49 age-matched typically developing (TD) boys. The cortical source activity was estimated using the beamformer approach and individual brain models. We then extracted the 1/f slope by fitting a linear function to the log–log-scale power spectra in the high-frequency range.

**Results:**

The global 1/f slope averaged over all cortical sources demonstrated high rank-order stability between the two conditions. Consistent with previous research, it was steeper in the eyes-closed than in the eyes-open condition and flattened with age. Regardless of condition, children with ASD and below-average IQ had flatter slopes than either TD or ASD children with average or above-average IQ. These group differences could not be explained by differences in signal-to-noise ratio or periodic (alpha and beta) activity.

**Limitations:**

Further research is needed to find out whether the observed changes in E–I ratios are characteristic of children with below-average IQ of other diagnostic groups.

**Conclusions:**

The atypically flattened spectral slope of aperiodic activity in children with ASD and below-average IQ suggests a shift of the global E–I balance toward hyper-excitation. The spectral slope can provide an accessible noninvasive biomarker of the E–I ratio for making objective judgments about treatment effectiveness in people with ASD and comorbid intellectual disability.

**Supplementary Information:**

The online version contains supplementary material available at 10.1186/s13229-022-00498-2.

## Background

The hypothesis of an altered ratio between neural excitation and inhibition (E–I ratio) as a probable cause of autism was first introduced by Rubenstein and Merzenich in 2003 [1] and received numerous confirmations in genetic and animal research [2]. Indeed, the majority of the animal models of autism spectrum disorder (ASD) target functioning of the excitatory or inhibitory neurons and alter the balance of their activity [3, 4], while pharmacological drugs that correct this balance can help to rescue the disease phenotype, at least in experimental animals [4]. Although Rubenstein and Merzenich initially related ASD to an elevated E–I ratio, later it became clear that some forms of ASD can be characterized by decreased E–I ratio [5, 6] and that alternations in the E–I ratio can be region-specific, reflecting homeostatic regulation of local E–I imbalances [7, 8]. Still, there is evidence that, in many cases, the global deficit associated with ASD can be characterized as predominant neuronal hyper- [9–11] or hypo [5, 6]-excitability. Therefore, despite simplification, the concept of a global E–I balance is important for understanding the pathophysiological mechanisms associated with ASD [2].

The genes that lead to the E–I imbalance through their effect on neurotransmission are often implicated in both ASD and intellectual disability [12–14]. People with ASD and comorbid intellectual disability are more likely to have epilepsy than autistic individuals without intellectual disability [15, 16], possibly because of more severe disturbances of the E–I balance. Although such patients may benefit most from targeted pharmacological treatment, neuroimaging studies looking for ‘autism biomarkers’ rarely include individuals from the lower end of the intelligence quotient (IQ) range [17].

Physiological parameters that quantify the degree and direction of the E–I imbalance in individuals with ASD—particularly in those with below-average IQ—could provide valuable biomarkers for translational research, stratifying patients for clinical trials, and making objective judgments about treatment efficacy [2].

Brain signals recorded with magnetoencephalography (MEG) directly reflect the activity of cortical neuronal populations and could potentially help to estimate the E–I balance globally as well as in distinct cortical regions. The power spectrum of electrophysiological signals measured with MEG or electroencephalography comprises the ‘periodic’ part that reflects rhythmic brain activity and the ‘aperiodic’ part—the ‘neural noise’ [18, 19]. The power of the aperiodic component falls off with increasing frequency following a power-law functional relationship, which is reflected in a linear relationship on a logarithmic scale. The slope of this linear function becomes flatter (less negative) during maturation [20–25], reflecting the increasing complexity of neural networks and/or changes in their structure and neurochemistry. At the same time, the spectral slope is sensitive to changes in excitatory and inhibitory neurotransmission during low arousal states, e.g., anesthesia and sleep (when the decrease in the E–I ratio is indexed by a steeper slope) [26–30] and may reflect E–I imbalance in neuropsychiatric disorders [31]. It has been argued that the flattened spectral slope may reflect an elevated background rate of neurons’ firing, which is decoupled from an oscillatory carrier frequency and is driven by an increased E–I ratio (i.e., ‘noise’) [19, 27].

Although a spectrum of resting-state neural activity can be separated computationally into periodic and aperiodic components, the estimation of the aperiodic 1/f slope in the presence of oscillatory activity may vary depending on the method used, frequency bandwidth chosen, etc. [32, 33]. To overcome these obstacles, the 1/f spectral slope can be estimated in ~ 30–70 Hz range, where rhythmic activity is usually absent [27, 29, 32].

Here, we used MEG and individual magnetic resonance imaging (MRI)-based brain models to capture the spectral slope from high-frequency brain activity measured ‘at rest’ in children with ASD and below-average IQ, those with average IQ, and in age-matched TD children. There is strong evidence that brain structure and functioning, including the E–I balance, may be differently affected in males and females with ASD [34, 35]. The relatively small sample size in our neurophysiological study does not allow examining gender differences in E–I balance. Therefore, here we focused on boys, who are about 4 times more likely to be diagnosed with ASD than girls [36].

We predicted that boys with ASD and low IQ would yield flatter slopes of the aperiodic component of the power spectra in the high-frequency range (35–45 Hz) than the other two groups of boys, indicative of their globally disturbed (predominantly elevated) E–I ratios.

## Methods

### Participants

The participants were 49 boys with ‘non-syndromic’ ASD and 49 TD boys aged 6–15 years (see Additional file 1: Methods for recruitment and exclusion details). Each participant in the ASD group had a diagnosis of ASD confirmed by a child psychiatrist according to the DSM-5 criteria and interviews with children’s parents or caregivers. Parents/caregivers of all children were asked to fill in the Russian version of the Social Responsiveness Scale for children (SRS) [37].

IQ has been evaluated through standard scores on K-ABC subscales, as well as by calculating the Mental Processing Index (MPI) [38]. MPI assessment requires minimal use of verbal content, and this index is well suited both for TD children and for participants with atypical cognitive functioning, such as ASD [39]. The MPI includes four global scales: (1) the Sequential Processing scale, which assesses a child’s ability to arrange linearly or temporally related units of information in sequential or serial order, (2) the Simultaneous Processing scale, which involves abilities for holistic synthesis and integration of visual information, (3) the Learning scale, which evaluates a child’s ability to store and efficiently retrieve newly or previously learned information, (4) the Planning Scale, which focuses on Pattern Reasoning and Story Completion abilities. Scale scores are presented as age-adjusted standardized scores, normalized so that the mean is 100 and the standard deviation is 15, which is consistent with most cognitive assessment instruments. In the KABC-II, the term ‘average’ is used for MPI scores from 85 to 115, ‘above average’ for scores 116 to 130, ‘below average’ for scores 69 to 84, and ‘lower extreme’ for scores below 69. We used MPI scores to divide ASD participants into groups according to their cognitive abilities and to assess correlations with neurophysiological variables.

The study has been approved by the Ethical Committee of the Moscow State University of Psychology and Education. Verbal assent to participate in the experiment was obtained from all the subjects; parents/caregivers provided informed written consent.

For all the participants, the MEG data (see below) were available in the ‘eyes open’ (EO) condition, and for 45 TD and 38 ASD children the data were also available in the ‘eyes closed’ (EC) condition. The TD and ASD participants did not differ in age (Mann–Whitney *U* test, EO condition: *N*_TD_ = 49, *N*_ASD_ = 49, *U* = 1014.5, *Z* = 1.31, *p* = 0.19; EC condition: *N*_TD_ = 45, *N*_ASD_ = 38, *U* = 745.0, *Z* = 1.15, *p* = 0.25), but differed in the MPI scores (Mann–Whitney *U* test, *p* < 10^–10^ for both conditions). The SRS scores were significantly higher in participants with ASD than in the control sample (Mann–Whitney *U* test, *p* < 10^–10^ for both conditions).

The ASD participants were further subdivided into two groups based on their MPI IQ scores: *average IQ* (MPI > 85; ASD_>85_) and *below-average IQ* (MPI < 85; ASD_<85_). ASD children with IQ above 85 still had lower IQ than TD children (*T* test, *p* < 10^–8^ for subjects in both EO and EC conditions). The SRS scores were significantly higher in ASD than TD participants (*T* test, *p* < 10^–10^ for subjects in both EO and EC conditions, TD vs. ASD_>85_ and TD vs. ASD_<85_) and tended to be higher in ASD_<85_ group than in ASD_>85_ group (*T* test, EO: *N*_ASD>85_ = 27, *N*_ASD<85_ = 18, *t*_(43)_ = 1.71, *p* = 0.09; EC: *N*_ASD>85_ = 20, *N*_ASD<85_ = 14, *t*_(32)_ = 1.78, *p* = 0.08), but age did not differ in these three groups (Mann–Whitney *U* test, *p*’s > 0.3 for both conditions).

Detailed information about the experimental samples is summarized in Table 1. The number of participants of different age ranges is given in Additional file 1: Table S1.

**Table 1:** Characteristics of the experimental samples

Sample	TD	ASD_>85_	ASD_<85_
Eyes-open condition			
*N* subjects	49	30	19
Age, years	10.81 ± 2.37	10.29 ± 2.95	10.57 ± 2.14
MPI IQ	121.57 ± 12.19	100.37 ± 12.31	69.26 ± 9.36
SRS^1^	42.1 ± 22.04 (*N* = 48)	98.22 ± 23.98 (*N* = 27)	110.06 ± 20.57 (*N* = 18)
Eyes-closed condition			
*N* subjects	45	23	15
Age, years	10.73 ± 2.30	10.73 ± 3.12	10.05 ± 2.02
MPI IQ	120.78 ± 12.16	99.43 ± 12.97	71.60 ± 8.77
SRS	41.3 ± 22.16 (*N* = 45)	97.65 ± 25.27 (*N* = 20)	111.85 ± 18.69 (*N* = 14)

TD, typically developing children; ASD, children with autism spectrum disorder; MPI, Mental Processing Index; ASD_>85_, children with ASD and MPI above 85; ASD_<85_, children with ASD and MPI below 85; and SRS, Social Responsiveness Scale

^1^The number of participants for whom information on the SRS questionnaire was available is given in parentheses

### MRI data acquisition and processing

Structural magnetic resonance (MR) scans (voxel size 1 mm × 1 mm × 1 mm) were performed on a General Electric Signa 1.5 T scanner. The T1 images were processed using the default FreeSurfer (v. 6.0.0) ‘recon-all’ pipeline. For the MEG analysis, the cortical surface was parcellated into 448 similar-size labels, as described by Khan et al. [40].

### MEG data acquisition and preprocessing

MEG was recorded with a 306-channel MEG system (Vectorview, Elekta-Neuromag). The ‘resting state’ recordings were always performed in the beginning of the recording session followed by experimental tasks analyzed elsewhere [41, 42]. The participants were instructed to sit calmly, first with eyes closed, and then, they were instructed to open their eyes. No other instructions were given. Two to four minutes of MEG were recorded in each experimental condition. Participants’ behavior was constantly monitored through a video camera positioned inside the MEG room. Trigger marks and experimenter’s comments were further used to select MEG intervals for analysis.

The signal was initially sampled at 1000 Hz with a 0.03–300 Hz band-pass filter. Head position was continuously monitored and corrected to each subject’s initial head position using MaxFilter software (v. 2.2). The temporal signal-space separation (tSSS) method [43] with correlation limit 0.9 was applied to compensate for correlated environmental noises.

Further steps of data preprocessing were performed with the MNE-python toolbox (v. 0.22.0) [44]. The raw data were down-sampled to 500 Hz. The signal-space projections method [45] was applied to the raw data to suppress biological artifacts (eye blinks and cardiac artifacts). The raw signal was subdivided into 1 s non-overlapping epochs. Epochs contaminated with bursts of myogenic artifacts were automatically detected using MNE-python function ‘annotate_muscle_zscore’ with default parameters (ch_type =  ‘mag,’ threshold = 5.0, min_length_good = 0.2, filter_freq = [110, 140]) and excluded from analysis. From this point on, only gradiometers were used for analysis. Those epochs where the head origin deviated from the initial position by more than 20 mm were excluded from analysis. The percent of dropped epochs was higher in the ASD than in TD children in the EO condition (Mann–Whitney *U* = 940.0, *p* = 0.03) and tended to be higher in the EC condition (Mann–Whitney *U* = 733.0, *p* = 0.10). To preclude group differences in timing of the analyzed epochs relative to the condition onset, we randomly excluded 10% of the ‘good’ epochs in the TD group and then have chosen the first 60 epochs per condition and subject in both ASD and TD groups for further analysis. As a result, there was no significant difference in epoch times relative to the condition onset time between the three experimental groups for both EO (Kruskal–Wallis one-way analysis of variance (ANOVA), H(2, *N* = 98) = 2.73, *p* = 0.25) or EC (Kruskal–Wallis one-way ANOVA, H(2, *N* = 83) = 2.38, *p* = 0.30) conditions. The mean distance of the head origin from the initial position did not differ between the groups (Kruskal–Wallis one-way ANOVA: EO, H(2, *N* = 98) = 2.58, *p* = 0.27, mean distance in TD/ASD_>85_/ASD_<85_ was 4.1/4.8/5.3 mm; EC, H(2, *N* = 83) = 0.58, *p* = 0.75, mean distance in TD/ASD_>85_/ASD_<85_ was 2.5/2.8/2.6 mm).

### Empty room recording

To estimate background noise, MEG data were recorded in the absence of a subject (empty room), directly before each subject’s MEG recording. The empty room MEG recordings were spatially filtered using the tSSS method [43] and were further used to estimate the background noise in the frequency range of interest, as well as to construct the noise covariance for the standardized low-resolution brain electric tomography (sLoreta) analysis [46], as described below.

### Choice of the frequency range for the spectral slope estimation

**Fig. 1:**
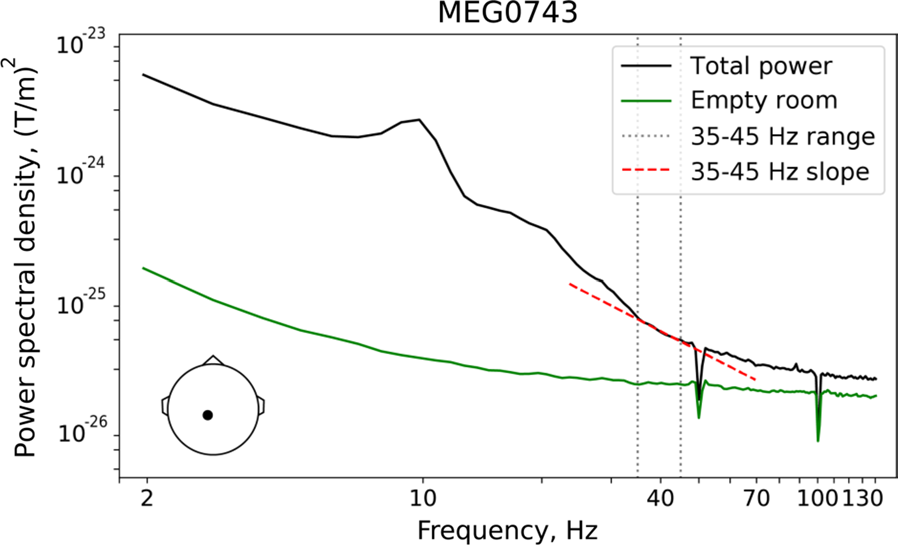
Spectral analysis for a single central gradiometer (average over all participants). The gradiometer is positioned at a large distance from the cranial muscles; position of the gradiometer is shown in the lower left corner. Total magnetoencephalographic (MEG) power in the eyes-open condition is plotted in black, and the empty room noise recording is plotted in green. Spectral peaks (i.e., periodic signals) are absent in the 35–45 Hz range (marked by gray dotted lines), and the spectral power declines approximately linearly in this frequency range (red dashed line). The power spectra are plotted on a logarithmic scale

Following previous studies [27, 29, 32], we sought to estimate the slope of aperiodic activity at the high-frequency part of the spectrum. To choose the optimal high-frequency range for the aperiodic slope analysis, we first estimated the power spectral density (PSD) in the sensor space using Welch’s method. Figure 1 shows an averaged over all participants power spectrum of a centrally located gradiometer (‘total power’) and the grand averaged power spectrum of the signal recorded by this gradiometer in the empty room.

The overall ‘total power’ spectrum is characterized by presence of peaks corresponding to the typical neuro-oscillation bands (alpha, beta). For frequencies above approximately 35 Hz, peaks are not obvious and the 1/f-like trend is apparent. We therefore set the lower bound for our aperiodic activity slope estimate to 35 Hz. On the other hand, a knee appears in the spectrum for frequencies above approximately 50 Hz where the ‘total power’ approaches that of the empty room spectrum (i.e., where power is approximately constant as a function of frequency). In order to also avoid potential effects of the 50 Hz power line cycle and the notch filter used for its removal, we set the upper bound for our aperiodic activity slope estimate to 45 Hz. In the 35–45 Hz range, the grand averaged over all subjects in EO condition spectrum displays a clear 1/f-like characteristic (i.e., it is well approximated by a line on the log–log scale; Fig. 1).

### Estimation of the spectral slope in the source space

We performed source localization analysis using linearly constrained minimum variance (LCMV) beamformer spatial filters [47] that significantly reduce the contribution of biological artifacts (e.g., myogenic activity) into the brain activity estimated at the source level [48]. In order to gauge the effect of residual myogenic contamination on the group differences in spectral slope, we also compared the LCMV results with those obtained using sLoreta [46]. In contrast to beamforming methods, the methods based on the least-squares minimum norm algorithms such as sLoreta tend to overfit non-brain signals [49]. Therefore, the contribution of myogenic artifacts to the activity estimated at cortical sources using sLoreta is expected to be greater than in the case of LCMV beamformer (see Additional file 1: Results: ‘Contribution of myogenic artifacts to the group differences in spectral slope’).

MEG data were co-registered with individual’s MR image of the head, and the forward model was created using a one-layer boundary element model (BEM) and surface-based source space (4096 vertices per hemisphere). The raw data were filtered between 30 and 140 Hz, and the data covariance matrix was estimated individually for each condition. The LCMV beamformer filter with a regularization parameter of 0.1 was calculated for the source orientation that maximizes power. The same filter was applied to each 1 s data epoch. We then calculated the neural activity index (NAI) that represents the signal normalized to the spatially inhomogeneous noise [47]. In each source vertex, the spectral power was estimated using Welch’s method. The power spectra at each vertex were normalized by the maximum value at the corresponding spectra and then averaged within each of 448 labels. For each label, we then fitted a linear regression line to the logarithm of the power spectrum between 35 and 45 Hz vs. the logarithm of the frequency (‘polyfit’ function, Python library ‘NumPy,’ v. 1.20.2). The linear term coefficient was used as an estimate of the 35–45 Hz spectral slope (hereinafter referred to as the ‘spectral slope’).

We also estimated the aperiodic slope in the 2–45 Hz range using the ‘FOOOF’ parameterization algorithm [50]. A detailed description and discussion of these results are given in Additional file 1: Results (‘Spectral slope estimated with FOOOF’).

### Estimation of the periodic power in the alpha and beta frequency ranges

While beamformers provide good estimates of spectral power changes (e.g., when contrasting conditions) [51], they might provide suboptimal estimates of the *absolute* power. Therefore, to quantify the periodic spectral power, we performed source localization with sLoreta that has zero localization error [46]. For this analysis, MEG and empty room signals were band-passed in the 1–47 Hz range. The noise covariance matrix was derived from one minute of ‘MaxFiltered’ empty room data. The sLoreta inverse solution was estimated with the following parameters: semi-orthogonal orientation of the dipole source to the cortical surface (parameter loose set to 0.4), depth weighting parameter set to 0.8, and signal-to-noise ratio (SNR) set to 1. The inverse operator was applied to each one-second data epoch, and the power spectra were obtained at the source level using Welch’s method. To separate the periodic from aperiodic spectral power in the alpha and beta bands, we applied the FOOOF algorithm [50] to the average power spectra of each label in the 2–45 Hz range. The periodic component was then estimated as: [original spectra—aperiodic fit]. The mean alpha and beta powers were assessed as an average spectral power of the periodic component in the 7–13 Hz and 14–25 Hz bands, respectively. This approach gives a reasonable approximation on the power of periodic component in alpha and beta ranges (see ‘Results’ section).

### Estimation of the sensitivity maps

To ensure that our results were not biased by head size, we calculated individual ‘sensitivity maps’ (‘mne.sensitivity_map’ function in MNE-python), which estimate how well each source is sampled by a sensor array (i.e., the planar gradiometers). The mean sensitivity in a label was then used as a nuisance variable in our regression analysis.

### Statistical analysis

To estimate the rank-order stability of the spectral slopes, we calculated intraclass correlation coefficients (ICC) between conditions (EO/EC). ICC was estimated for a fixed set of ‘raters’ (EO, EC) and for the average of all ‘ratings’ (mean spectral slopes) [52].

The mean periodic alpha and beta spectral power values were log10-transformed to normalize the distributions. Because distributions of the analyzed variables (log10-transformed alpha and beta power, mean slope) did not significantly differ from normal in either EO or EC conditions (Shapiro–Wilk test, all *p*’s > 0.25), to test for the group differences we used parametric analysis of covariance (ANCOVA) with age as the covariate.

We calculated Spearman correlation coefficients to estimate the relationship between spectral slope and IQ, SRS, and age, because we expected a monotonic, but not necessarily strictly linear relationships. To control for nuisance variables (background noise, sensitivity, spectral power, age), we calculated partial Spearman correlations.

The Benjamini–Yekutieli false discovery rate (FDR) correction for multiple comparisons was applied to the *p* values when tests were calculated for multiple cortical labels. The threshold 0.05 was applied to both the univariate and FDR-adjusted *p* values.

## Results

### State dependency and rank-order stability of the spectral slope

To become a clinically useful signature of E–I balance, the spectral slope must not only be sensitive to changes in the neural E–I ratio, but also reflect trait-like individual differences that remain stable across different functional states. In awake humans, the E–I balance is shifted toward higher inhibition during states associated with high alpha power [53–55]. We therefore expected that if the spectral slope in children with and without ASD is sensitive to changes in the E–I ratio, then it would be steeper (more negative) in the EC condition (characterized by stronger alpha activity), as compared with the EO condition. We also proposed that if the spectral slope represents a stable individual characteristic, it should have high rank-order stability between the EO and EC conditions.

**Fig. 2:**
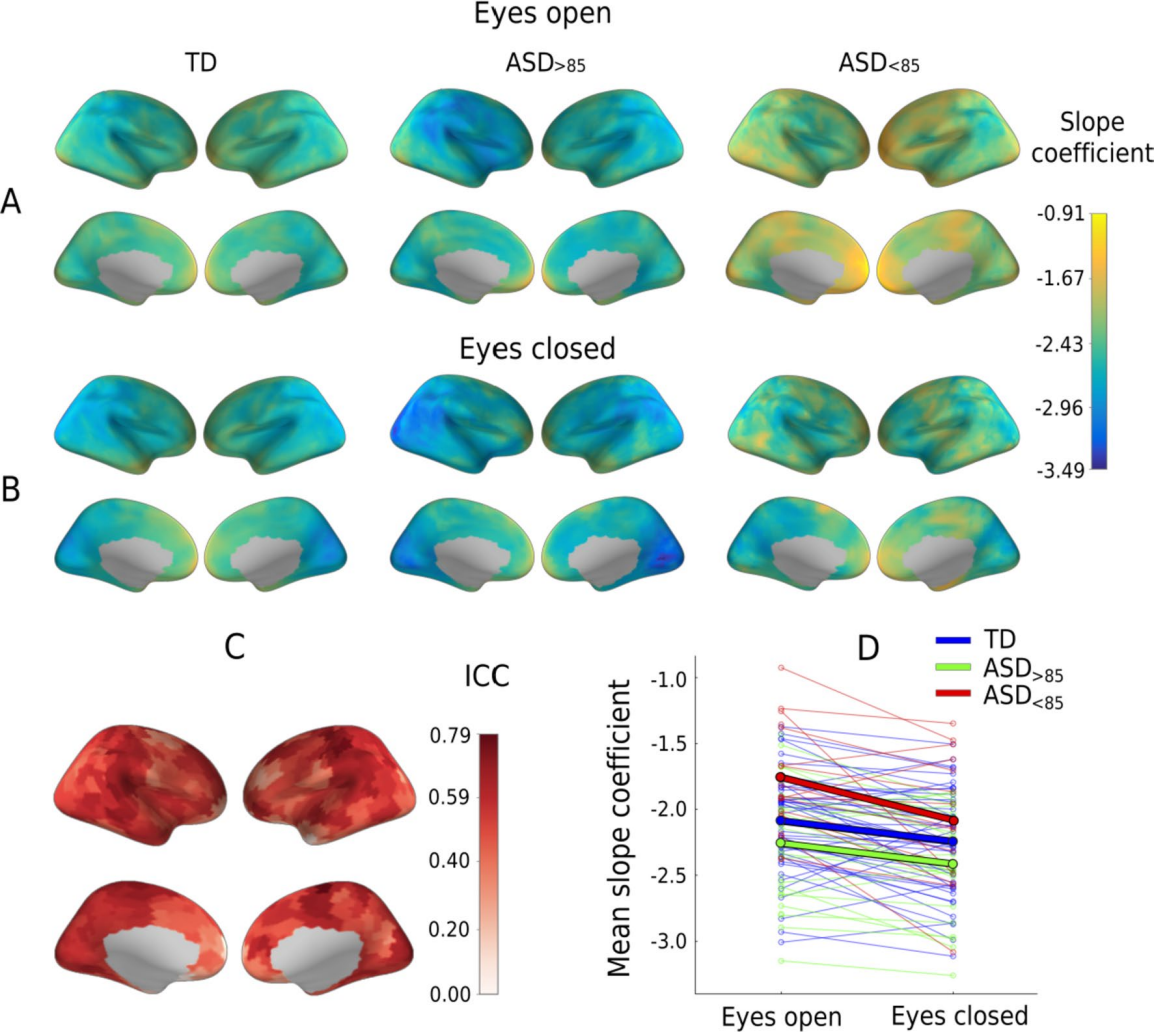
Spectral slope: effect of experimental condition and rank-order stability. **(A, B)** Cortical distribution of the 35–45 Hz spectral slope coefficients in the three groups of participants in the eyes-open (A) and eyes-closed (B) conditions. The activity of the cortical sources was estimated using the linearly constrained minimum variance (LCMV) beamformer method. **(C)** Rank-order stability of the 35–45 Hz spectral slope between the eyes-open and the eyes-closed conditions: cortical distribution of intraclass correlation coefficients (ICC) for 83 participants for whom data from both conditions were available. **(D)** 35–45 Hz spectral slope averaged over all cortical sources in the two experimental conditions. Thin lines show individual subjects; thick lines—the group average. Colors correspond to different groups of participants. TD, typically developing children; ASD, autism spectrum disorder; ASD_>85_, children with ASD and Mental Processing Index above 85; and ASD_<85_, children with ASD and Mental Processing Index below 85

Figure 2A, B shows the cortical distribution of the spectral slopes in the three groups of children. Using ICC, we estimated the rank-order stability of the spectral slopes of the separate cortical regions (448 labels) between the EC and EO conditions. We also estimated the rank-order stability of the spectral slope averaged over all cortical labels (hereafter, the mean slope). The rank-order stability of the mean slope was good (ICC_(82)_ = 0.87, *p* < 0.000001) [52] and exceeded that in the individual cortical labels, where it ranged from 0.06 to 0.79 (Fig. 2C). Considering the highest rank-order stability of the mean spectral slope, we used this measure for further analysis.

A paired t test revealed a highly significant and strong decrease in the mean slope from the EO to EC condition in the combined group of subjects (*T*_(82)_ = 5.81, *p* < 0.000001, Cohen *d* = 0.64), as well as in the separate groups (TD: *T*_(44)_ = 3.67, *p* = 0.0006, Cohen *d* = 0.55; ASD_>85_: *T*_(22)_ = 3.38, *p* = 0.0003, Cohen *d* = 0.71; ASD_<85_: *T*_(14)_ = 3.31, *p* = 0.005, Cohen *d* = 0.85; Fig. 2D).

### Effect of age, condition, IQ, and autism diagnosis on the mean spectral slope

**Fig. 3:**
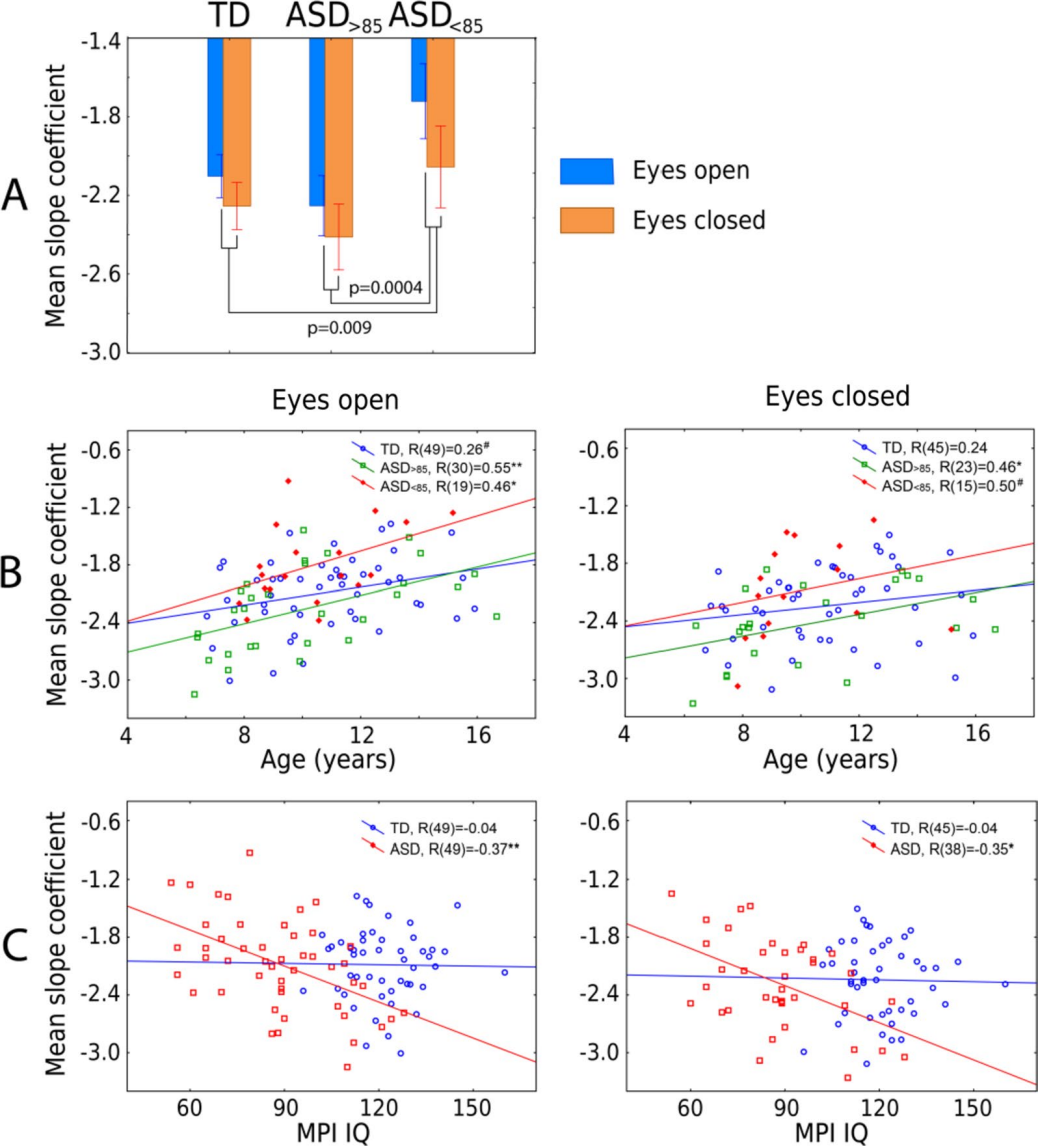
Effect of age and MPI IQ on the mean spectral slope. **(A)** Difference in the mean spectral slope between the three groups of participants. Vertical bars denote 0.95 confidence intervals. The *p* values (planned comparisons, two-sided t test) are given for between group comparisons. (Eyes-open and eyes-closed conditions were pooled.) **(B)** Age-related changes in the mean spectral slope in the three groups of participants. *R*’s denote the Spearman correlation coefficients. The group differences between correlation coefficients (TD/ASD_<85_, TD/ASD_>85_, ASD_>85_/ASD_<85_) are not significant (all *p*’s > 0.073). **(C)** The relationship between MPI IQ and mean spectral slope in TD children and ASD children. *R*’s denote the partial Spearman correlation coefficients, adjusting for age. TD, typically developing children; ASD, autism spectrum disorder; MPI, Mental Processing Index; ASD_>85_, children with ASD and MPI above 85; and ASD_<85_, children with ASD and MPI below 85

The mean spectral slope values for the three groups of participants and the two experimental conditions are shown in Fig. 3A. The general linear model analysis with factors Group, Condition, and Age revealed a main effect of Age (*F*_(1,79)_ = 12.76, *p* = 0.0006, *η*^2^ = 0.14) indicating a general flattening of the spectral slope with brain maturation (Fig. 3B). The highly significant main effect of Condition (*F*_(2,79)_ = 38.3, *p* < 1e−10, *η*^2^ = 0.33) did not interact with Group (Condition × Group: *F*_(2,79)_ = 2.4, *p* = 0.1, *η*^2^ = 0.06) or Age (Condition × Age: *F*_(2,79)_ = 2.6, *p* = 0.1, *η*^2^ = 0.03), thus suggesting that the strong changes in spectral slope associated with opening/closing the eyes are comparable across age and in groups from different diagnostic categories. As expected, we found a significant main effect of Group (*F*_(2,79)_ = 6.95, *p* = 0.0017, *η*^2^ = 0.15): The slope was flatter in the ASD_<85_ than in either TD (*F*_(1,79)_ = 7.2, *p* = 0.009) or ASD_>85_ (F_(1,79)_ = 13.8, *p* = 0.0004) groups. Unexpectedly and by contrast, though, the slope for the ASD_>85_ group tended to be steeper than that in the TD group (*F*_(1,79)_ = 2.80, *p* = 0.098).

We repeated the analysis with factors Group and Age for the EO condition for which the data were available for a greater number of participants. Again, there was a significant effect of Age (*F*_(1,94)_ = 18.1, *p* = 0.00005, *η*^2^ = 0.16) and Group (*F*_(2,94)_ = 8.4, *p* = 0.00045, *η*^2^ = 0.15), which was explained by a flatter slope in the ASD_<85_ group than in the rest of participants.

To test whether there is a link between IQ and the mean spectral slope in TD children, and whether it differs from that in children with ASD, we calculated correlations between the spectral slope and IQ in the TD and combined ASD groups. The ASD vs. TD difference in partial Spearman correlation coefficients (when adjusting for age) was significant for the EO condition (*N*_TD_ = 49, *N*_ASD_ = 49, *Z* = 1.67, *p* = 0.047), and the same tendency was observed for the EC condition (*N*_TD_ = 45, *N*_ASD_ = 38, *Z* = 1.41, *p* = 0.079). While children with ASD demonstrated reliable negative correlations between the mean slope coefficient and IQ, no such correlations were found in the TD group (Fig. 3C).

We also tested for the correlation between the mean slope and SRS scores in the ASD groups. In children with ASD, there was a tendency for correlation between MPI IQ and SRS scores: *N* = 45, Spearman *R* = − 0.28, *p* = − 0.06). However, the correlations between the mean slope and SRS scores were not significant (EO: *N*_ASD_ = 45, *R* = 0.14, *p* = 0.34; EC: *N*_ASD_ = 34, *R* = 0.29, *p* = 0.1).

### Cortical distribution of the correlations between spectral slope and IQ in children with ASD

**Fig. 4:**
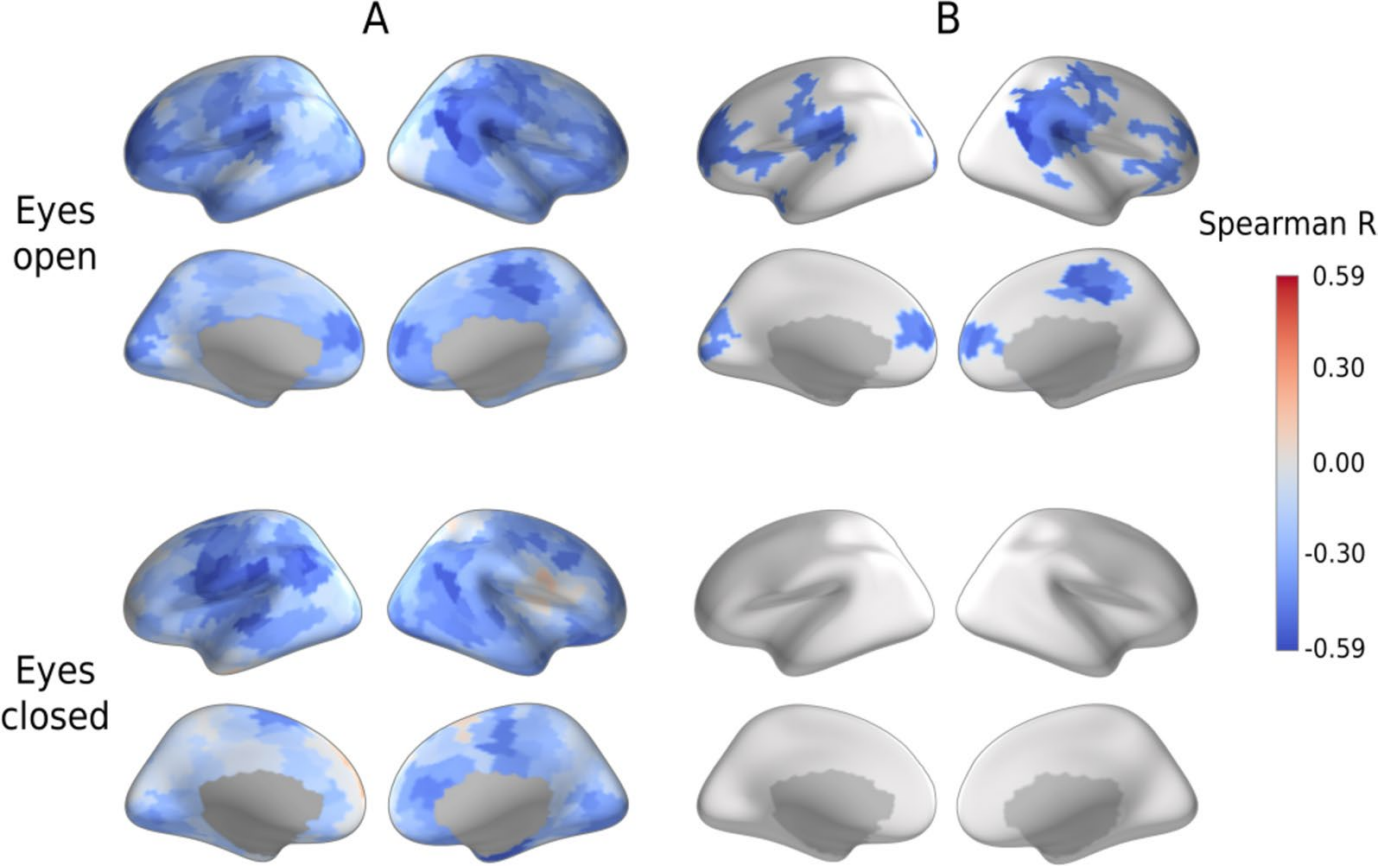
Partial Spearman correlations between spectral slopes and Mental Processing Index (MPI IQ). Correlations are shown in the individual cortical labels in children with autism spectrum disorder (ASD) in the eyes-open and eyes-closed conditions, adjusting for age and label-specific sensitivity. **(A)** Uncorrected for multiple comparisons. **(B)** False discovery rate corrected

To ensure that the link between the mean spectral slope and IQ is not driven by brain regions wherein source estimates are most susceptible to myogenic contamination, we calculated partial Spearman correlation coefficients between MPI IQ and spectral slopes in the separate cortical labels, while controlling for age and sensitivity (Fig. 4A; see Methods for details). For the EO condition, the largest group of significant correlations (*p* < 0.05, FDR corrected) overlapped the supramarginal area and temporoparietal junction of the right hemisphere (Fig. 4B, see Additional file 1: Table S2 for the full list of the cortical labels that survived the FDR correction). In general, the spatial distribution of the correlations is evidence against a potential ‘myogenic explanation’ of their origin. Similar negative correlations were observed for the EC condition (Fig. 4A), but none of them survived correction for multiple comparisons (Fig. 4B).

### Spectral slope and periodic activity in the alpha and beta range

It has been argued that changes in the spectral slope of brain activity are secondary to the changes in periodic activity (e.g., power of alpha or beta rhythms) [56]. The power of periodic activity varies with functional and behavioral state, which in turn may depend on a subject’s diagnosis and IQ. To ensure that the group differences in the spectral slope were not explained by differences in the periodic activity, we estimated the periodic spectral power in the alpha and beta bands.

**Fig. 5:**
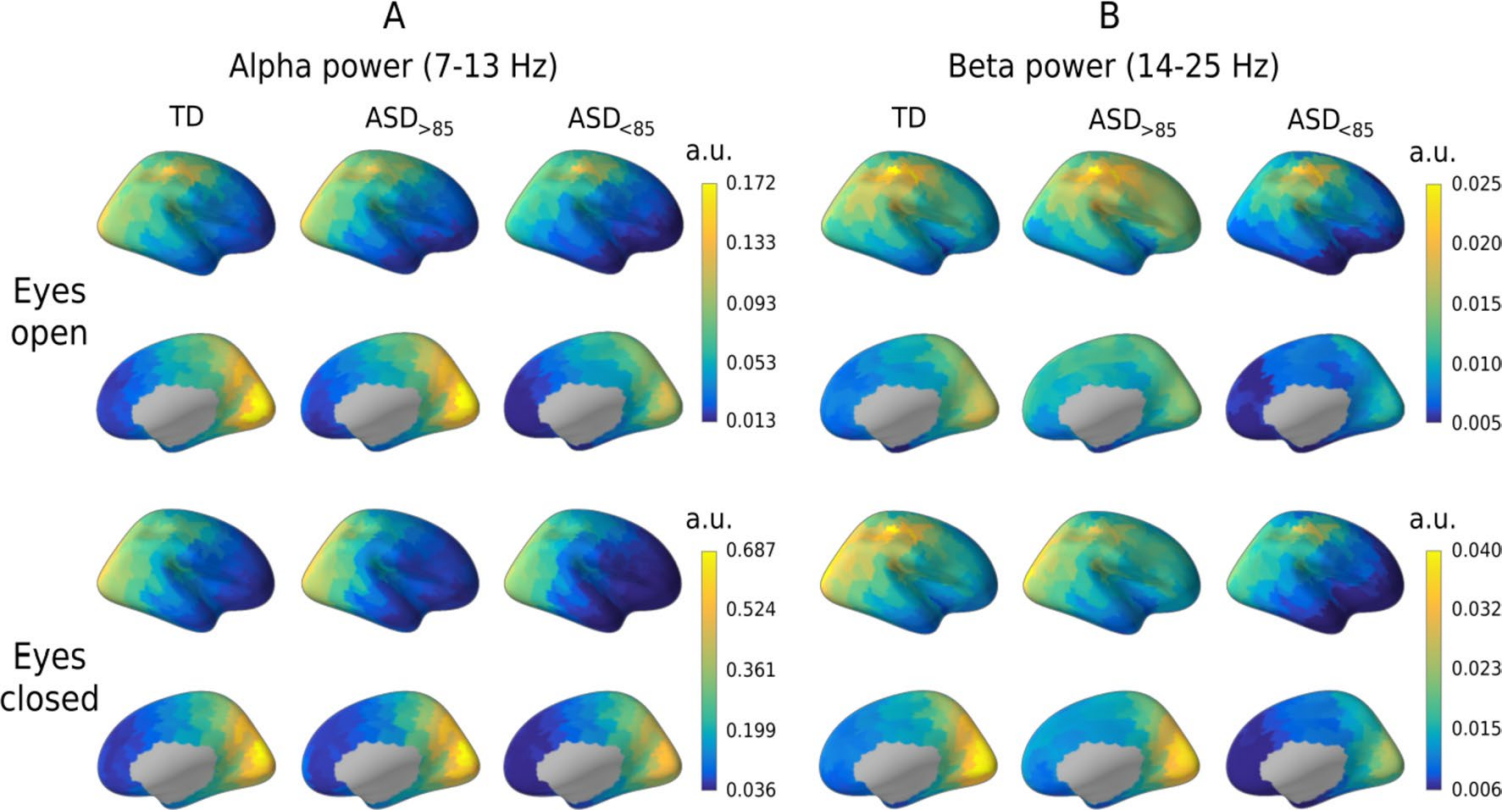
Cortical distributions of the power of periodic activity in the alpha and beta bands. Distributions are shown in the three groups of participants and in the eyes-open and eyes-closed conditions. Note that the scales differ for the bands and conditions. TD, typically developing children; ASD, autism spectrum disorder; ASD_>85_, children with ASD and Mental Processing Index above 85; and ASD_<85_, children with ASD and Mental Processing Index below 85; a.u., arbitrary units

Figure 5 shows cortical distributions of alpha and beta periodic power in the three groups of participants. In accord with the results of Muthukumaraswamy and colleagues [56], a stronger periodic alpha and beta power was associated with a steeper, more negative spectral slope in both TD and ASD participants and in both experimental conditions (Table 2). However, unlike the slope, the grand averaged periodic power did not differ between the groups (ANOVA Group effect for the alpha band, EO: *F*_(2,94)_ = 1.89, *p* = 0.16, *η*^2^ = 0.038; EC: *F*_(2,80)_ = 1.29, *p* = 0.28, *η*^2^ = 0.031; ANOVA Group effect for the beta band, EO: *F*_(2,94)_ = 1.4, *p* = 0.26, *η*^2^ = 0.028; EC: *F*_(2,80)_ = 2.1, *p* = 0.13, *η*^2^ = 0.049).

**Table 2:** Spearman correlations between the grand average spectral slope and power of periodic oscillations

	TD	ASD	TD + ASD
Eyes-open condition			
Alpha	*R*(49) = − 0.26^#^	*R*(49) = − 0.11	*R*(98) = − 0.19^#^
Beta	*R*(49) = − 0.36*	*R*(49) = − 0.36*	*R*(98) = − 0.36**
Eyes-closed condition			
Alpha	*R*(45) = − 0.57**	*R*(38) = − 0.25	*R*(83) = − 0.41**
Beta	*R*(45) = − 0.53**	*R*(38) = − 0.28^#^	*R*(83) = − 0.40**

Alpha, power of periodic oscillations in the alpha (7–13 Hz) band; Beta, power of periodic oscillations in the beta (14–25 Hz) band; TD, typically developing children; ASD, children with autism spectrum disorder; and TD + ASD, combined sample of the children

^#^*p* < 0.1; *p < 0.05; **p < 0.001

To further ensure that the link between spectral slope and IQ in the ASD group did not stem from inter-individual differences in periodic activity, we calculated partial Spearman correlations between the mean spectral slope and IQ while controlling for the mean alpha and beta power. Partialling out the periodic power did not significantly affect the correlation between the mean spectral slope and MPI IQ in children with ASD (EO: *N* = 49, *R*_partial_ = − 0.43, *p* = 0.003; EC: *N* = 38, *R*_partial_ = − 0.35, *p* = 0.04).

## Discussion

The power of electrophysiological brain activity exhibits a 1/f-like dependence, and the flattened slope of the power decay as a function of frequency has been previously linked to a shift in the neural E–I balance toward excitation [26–30]. Here, we found that the spectral slope (averaged over cortical regions) was flatter in children with ASD and below-average IQ than in either TD children or in those with ASD and average IQ (Fig. 3A). This finding suggests that the neuronal E–I balance in the brains of the below-average IQ children with non-syndromic ASD is shifted toward excitation at the global scale.

Since the balanced activity of excitatory and inhibitory neurons is necessary for normal development and functioning of the brain [57–59], changes of the E–I ratio may contribute to intellectual disability in ASD. Indeed, mutations in the genes important for neural communication and the E–I balance may lead to both autism and intellectual disability [12–14, 60, 61]. Moreover, the putative shift to neuronal hyper-excitability observed in children with below-average IQ in our study may explain the high prevalence of epilepsy at the low-IQ end of the autism spectrum [15, 16].

The correlation of the spectral slope with IQ characterized children with ASD, but not TD children (Fig. 3C). It is noteworthy that in the ASD group these correlations formed a large right-hemispheric cluster (Fig. 4), which included the temporoparietal junction and supramarginal gyrus areas previously implicated in the theory of mind [62, 63] and empathy [64], respectively. Functional abnormalities in these areas were frequently found in ASD in functional MRI studies [65–68]. Moreover, the whole set of regions where the flattened spectral slope is linked to poor performance IQ (Additional file 1: Table S3) essentially overlaps with the frontoparietal executive network, whose abnormality is critically involved in executive dysfunction and the profound decrease in adaptive functioning in ASD (for a meta-analysis of functional MRI findings see [69]). The spatial information in the present study, however, should be interpreted with caution given the unequal sensitivity of the MEG sensors to the activity of different cortical sources and the moderate intraclass correlations of the region-specific spectral slopes between experimental conditions (Fig. 2C).

No significant differences in the spectral slope were found between children with ASD and average IQ and those with typical development. Interestingly, children with ASD and IQ above 85 display a tendency to have a steeper mean spectral slope than the TD children. It is likely that in the less severely affected children with ASD, the E–I imbalances are compensated on the global scale (or even biased toward inhibition) due to homeostatic plasticity mechanisms [8].

In contrast to MPI IQ, SRS scores in children with ASD did not correlate significantly with mean spectral slope. Perhaps the global increase in the E–I ratio primarily affects general cognitive abilities, whereas social deficits may be caused by local E–I imbalances in key systems involved in social cognition.

Although the mean spectral slope statistically significantly differentiated children with ASD and intellectual disability from the rest of the sample, these children share some properties of 1/f slope that are also pertinent to both average-IQ children with ASD and their TD peers.

Firstly, the mean spectral slope becomes less negative with age in all groups of participants (Fig. 3B). The developmental flattening of the spectral slope has been previously observed in TD infants [25], children [20, 21], and adolescents [24] and may reflect protracted maturational changes that are common for both TD and ASD individuals. These maturational changes do not necessarily reflect changes in neural E–I balance, since the 1/f slope is sensitive to a multitude of processes [18] that include not only developmental changes in the number and composition of gamma-aminobutyric acid (GABA) and glutamatergic receptors [70, 71], but also axon myelination [72] and changes in cortical thickness [73]. Considering the strong age dependence of the spectral slope, age should be carefully controlled for in studies employing the slope as a potential biomarker of the E–I balance.

Secondly, in both TD and ASD the spectral slope was steeper in case of stronger alpha and beta oscillations (Table 2) and generally steeper during the EC condition associated with high power in the alpha rhythm (Fig. 2D). These findings are in line with a previous report [56] and may stem from a partial overlap in the inhibitory mechanisms affecting alpha oscillations and the spectral slope. Indeed, ‘alpha states’ index reduced cortical excitability [53, 54] and are dominated by currents and firing in supragranular cortical layers [74], which display steeper 1/f slope than the other cortical layers, presumably due to longer time constants and a higher density of inhibitory transmembrane currents [75]. Therefore, inhibitory currents in the supragranular layers may both promote alpha oscillations and enhance the 1/f power decay. However, partialling out the variability in periodic power did not change the negative correlation of the spectral slope with IQ in ASD. This finding clearly indicates that the spectral slope brings unique information that is different from that which can be derived exclusively from the periodic activity.

Importantly, the correlations of the spectral slope with IQ in our study are unlikely to be explained by differences in biological or instrumental noise (see Additional file 1: Results for an analysis of the contribution of instrumental noise and muscle artifacts to the spectral slope and power in the 35–45 Hz range). Therefore, the link between IQ and the slope of the aperiodic component of the MEG-detected neural activity spectrum in children with ASD seems to be a true finding that reflects a globally elevated E–I ratio associated with intellectual disability that often co-occurs with autism.

It is important to note that the spectral slope is a relational measure and its absolute value is not particularly informative, since it depends on age and the method used to record neural activity (MEG, EEG, local field potential) [76], differs in different cortical areas [50], and—in case of EEG—depends on reference electrode [77]. Thus, a subject's spectral slope can provide information about neural E–I balance only when it is viewed in relation to the equivalent values in a normative sample.

Another important question is how the choice of the frequency range affects the spectral slope. With a rare exception [29], recent studies that used FOOOF [50] have estimated the aperiodic part of the spectrum in one broad frequency range, most often from delta to gamma frequency (~ 1–45 Hz) [20–25, 78–83]. Theoretically, aperiodic part of the spectrum is self-similar, or fractal, across a wide range of frequencies, such that the estimation of the spectral slope should be independent of the chosen fitting range [50, 84, 85]. In practice, however, the spectral slope may depend on the frequency range used [32]. In particular, FOOOF assumes all oscillation peaks lying within the fitting range because it does not fit partial Gaussian peaks. The delta activity is elevated in many developmental and psychiatric disorders [86–88], but detection of the delta peak can be difficult, which may lead to unrealistically negative slopes in subjects with elevated delta power [32]. To avoid this problem, in the present study we assessed the spectral slope in the high-frequency range, in which there are no spectral peaks. In the future, it would be important to investigate how the choice of frequency range affects estimation of the spectral slope.

Given the high cost of MEG, a question arises as to whether the spectral slope can be reliably estimated using a more affordable method, EEG. Bearing in mind the strong contamination of EEG by myogenic artifacts [89–91], and the likely differences in muscle tone between healthy controls and patients from clinical groups, caution should be exercised here. The spatial distribution of high-frequency EEG activity, spectral slopes, and their group differences may provide insight into the likely contribution of muscle artifacts to the experimental results. Perhaps spectral slopes can be most reliably estimated from EEG sensors located away from the cranial muscles (especially when using a bipolar reference or an averaged reference from a large number of EEG electrodes). Another way to reduce myogenic contamination in the EEG is by using source localization. As with MEG (Additional file 1: results; Additional file 1: Fig. S2), EEG source localization with beamformer techniques can potentially reduce the contribution of muscle artifacts to source activity in frontal, temporal, and occipital cortical areas.

### Limitations

An important limitation of our study is the high heterogeneity of our ‘idiopathic’ ASD sample that included subjects with different etiologies of ASD. To confirm the validity of the spectral slope biomarker, it would be important to investigate participants with monogenic disorders and animal models that are characterized by predominant shifts of the E–I balance toward excitation [9–11] or inhibition [5, 6, 78]. Another caveat is that putative abnormalities of the E–I ratio inferred here by the 1/f slope may not be specific to autism, but rather play a general role in the pathophysiology of intellectual disability. Future research should explore this question. It is also important to note that our study included a relatively small number of subjects, and they were all boys. Since the E–I balance may be differently affected in males and females with ASD [35], the possibility of generalizing our results to girls requires additional research.

There are also a few potential improvements that could increase the reliability of measurement of the 1/f spectral slope and promote its practical application. Firstly, while our participants were not engaged in any specific activity, one should consider using a modified experimental paradigm that allows for longer periods of artifact free data in children [92]. Secondly, the accuracy of the slope estimation could be improved by increasing the upper limit of the frequency range beyond 45 Hz, e.g., when the power line cycle is 60 Hz and/or a better SNR is available. Thirdly, the new and rapidly developing technology of ‘on-scalp’ MEG [93–95] may help to obtain equally high-quality data in all parts of the head, including the frontal areas, which are typically positioned furthest away from the sensors in ‘conventional’ MEG systems.

## Conclusions

In conclusion, the abnormally flattened 1/f spectral slope estimated in the high-frequency part of the MEG-detected neural activity spectrum is likely to reflect neuronal E–I imbalance associated with intellectual disability in children with ASD. Participants with below-average IQ are heavily underrepresented in autism research [96] and most neuroimaging studies only include high-functioning individuals with ASD [17]. Our MEG study demonstrates that it is feasible to collect resting-state MEG and structural MRI data in children with below-average IQ, and that cognitive ability, even though it is not a core aspect of the ASD diagnosis per se, should be considered as an important factor for research in the pathophysiology of this neurodevelopmental disorder. Our results indicate that the 1/f spectral slope estimated in the high-frequency part of the neural activity power spectrum may be a useful and objective biomarker of changes to the E–I ratio induced by pharmacological and other therapeutic interventions in low-functioning children with ASD.

## Supplementary Information

**Additional file 1.** Supplementary methods and results.

## Data Availability

The datasets used and/or analyzed during the current study are available from the corresponding author on reasonable request.

## Abbreviations

ANOVA: Analysis of variance
ANCOVA: Analysis of covariance
ASD: Autism spectrum disorder
ASD_>85_: Children with autism spectrum disorder and Mental Processing Index above 85
ASD_<85_: Children with autism spectrum disorder and Mental Processing Index below 85
E–I: Excitation–inhibition
EC: Eyes closed
EO: Eyes open
EEG: Electroencephalography
FDR: False discovery rate
GABA: Gamma-aminobutyric acid
ICC: Intraclass correlation coefficients
IQ: Intelligence quotient
LCMV: Linearly constrained minimum variance
MEG: Magnetoencephalography
MPI: Mental Processing Index
MRI: Magnetic resonance imaging
PSD: Power spectral density
sLoreta: Standardized low-resolution brain electric tomography
SNR: Signal-to-noise ratio
SRS: Social Responsiveness Scale
TD: Typically developing
tSSS: Temporal signal-space separation

## References

[1] Rubenstein JLR, Merzenich MM (2003). Model of autism: increased ratio of excitation/inhibition in key neural systems. Genes Brain Behav.

[2] Sohal VS, Rubenstein JLR (2019). Excitation–inhibition balance as a framework for investigating mechanisms in neuropsychiatric disorders. Mol Psychiatry.

[3] LeBlanc JJ, Fagiolini M (2011). Autism: a "critical period" disorder?. Neural Plast.

[4] Lee E, Lee J, Kim E (2017). Excitation/inhibition imbalance in animal models of autism spectrum disorders. Biol Psychiatry.

[5] Goncalves J, Violante IR, Sereno J, Leitao RA, Cai Y, Abrunhosa A (2017). Testing the excitation/inhibition imbalance hypothesis in a mouse model of the autism spectrum disorder: in vivo neurospectroscopy and molecular evidence for regional phenotypes. Mol Autism.

[6] Vogt D, Cho KKA, Lee AT, Sohal VS, Rubenstein JLR (2015). The parvalbumin/somatostatin ratio is increased in Pten mutant mice and by human PTEN ASD alleles. Cell Rep.

[7] Antoine MW, Langberg T, Schnepel P, Feldman DE (2019). Increased excitation–inhibition ratio stabilizes synapse and circuit excitability in four autism mouse models. Neuron.

[8] Nelson SB, Valakh V (2015). Excitatory/inhibitory balance and circuit homeostasis in Autism Spectrum Disorders. Neuron.

[9] Gibson JR, Bartley AF, Hays SA, Huber KM (2008). Imbalance of neocortical excitation and inhibition and altered UP states reflect network hyperexcitability in the mouse model of fragile X syndrome. J Neurophysiol.

[10] Rubinstein M, Westenbroek RE, Yu FH, Jones CJ, Scheuer T, Catterall WA (2015). Genetic background modulates impaired excitability of inhibitory neurons in a mouse model of Dravet syndrome. Neurobiol Dis.

[11] Sun AX, Yuan Q, Fukuda M, Yu W, Yan HD, Lim GGY (2019). Potassium channel dysfunction in human neuronal models of Angelman syndrome. Science.

[12] Betancur C, Sakurai T, Buxbaum JD (2009). The emerging role of synaptic cell-adhesion pathways in the pathogenesis of autism spectrum disorders. Trends Neurosci.

[13] Golden CEM, Buxbaum JD, Rubeis S (2018). Disrupted circuits in mouse models of autism spectrum disorder and intellectual disability. Curr Opin Neurobiol.

[14] Parikshak NN, Luo R, Zhang A, Won H, Lowe JK, Chandran V (2013). Integrative functional genomic analyses implicate specific molecular pathways and circuits in autism. Cell.

[15] Amiet C, Gourfinkel-An I, Bouzamondo A, Tordjman S, Baulac M, Lechat P (2008). Epilepsy in autism is associated with intellectual disability and gender: evidence from a meta-analysis. Biol Psychiatry.

[16] Capal JK, Carosella C, Corbin E, Horn PS, Caine R, Manning-Courtney P (2018). EEG endophenotypes in autism spectrum disorder. Epilepsy Behav.

[17] Jack A, Pelphrey KA (2017). Annual research review: understudied populations within the autism spectrum—current trends and future directions in neuroimaging research. J Child Psychol Psychiatry.

[18] Buzsaki G, Anastassiou CA, Koch C (2012). The origin of extracellular fields and currents - EEG, ECoG, LFP and spikes. Nat Rev Neurosci.

[19] Voytek B, Knight RT (2015). Dynamic network communication as a unifying neural basis for cognition, development, aging, and disease. Biol Psychiatry.

[20] Cellier D, Riddle J, Petersen I, Hwang K (2021). The development of theta and alpha neural oscillations from ages 3 to 24 years. Dev Cogn Neurosci.

[21] He W, Donoghue T, Sowman PF, Seymour RA, Brock J, Crain S, et al. Co-increasing neuronal noise and beta power in the 1 developing brain. Preprint at BioRxiv. 2019. 10.1101/839258.

[22] Hill AT, Clark GM, Bigelow FJ, Lum JAG, Enticott PG (2022). Periodic and aperiodic neural activity displays age-dependent changes across early-to-middle childhood. Dev Cogn Neurosci.

[23] Karalunas SL, Ostlund BD, Alperin BR, Figuracion M, Gustafsson HC, Deming EM (2021). Electroencephalogram aperiodic power spectral slope can be reliably measured and predicts ADHD risk in early development. Dev Psychobiol..

[24] McSweeney M, Morales S, Valadez EA, Buzzell G, Fox NA (2021). Longitudinal age- and sex-related change in background aperiodic activity during early adolescence. Dev Cogn Neurosci.

[25] Schaworonkow N, Voytek B (2021). Longitudinal changes in aperiodic and periodic activity in electrophysiological recordings in the first seven months of life. Dev Cogn Neurosci.

[26] Colombo MA, Napolitani M, Boly M, Gosseries O, Casarotto S, Rosanova M (2019). The spectral exponent of the resting EEG indexes the presence of consciousness during unresponsiveness induced by propofol, xenon, and ketamine. Neuroimage.

[27] Gao R, Peterson EJ, Voytek B (2017). Inferring synaptic excitation/inhibition balance from field potentials. Neuroimage.

[28] He BYJ, Zempel JM, Snyder AZ, Raichle ME (2010). The temporal structures and functional significance of scale-free brain activity. Neuron.

[29] Lendner JD, Helfrich RF, Mander BA, Romundstad L, Lin JJ, Walker MP (2021). An electrophysiological marker of arousal level in humans. Elife.

[30] Miskovic V, MacDonald KJ, Rhodes LJ, Cote KA (2019). Changes in EEG multiscale entropy and power-law frequency scaling during the human sleep cycle. Hum Brain Mapp.

[31] Molina JL, Voytek B, Thomas ML, Joshi YB, Bhakta SG, Talledo JA (2020). Memantine effects on electroencephalographic measures of putative excitatory/inhibitory balance in schizophrenia. Biol Psychiatry Cogn Neurosci Neuroimaging.

[32] Gerster M, Waterstraat G, Litvak V, Lehnertz K, Schnitzler A, Florin E, et al. Separating neural oscillations from aperiodic 1/f activity: challenges and recommendations. Neuroinform. 2022. 10.1007/s12021-022-09581-8. 10.1007/s12021-022-09581-8 PMC9588478 35389160

[33] Gyurkovics M, Clements GM, Low KA, Fabiani M, Gratton G (2021). The impact of 1/ f activity and baseline correction on the results and interpretation of time-frequency analyses of EEG/MEG data: a cautionary tale. Neuroimage.

[34] Lai MC, Lerch JP, Floris DL, Ruigrok ANV, Pohl A, Lombardo MV (2017). Imaging sex/gender and autism in the brain: etiological implications. J Neurosci Res.

[35] Trakoshis S, Martinez-Canada P, Rocchi F, Canella C, You W, Chakrabarti B (2020). Intrinsic excitation–inhibition imbalance affects medial prefrontal cortex differently in autistic men versus women. Elife.

[36] Zeidan J, Fombonne E, Scorah J, Ibrahim A, Durkin MS, Saxena S, Yusuf A, Shih A, Elsabbagh M. Global prevalence of autism: a systematic review update. Autism Res. 2022;1–13. 10.1002/aur.2696 PMC9310578 35238171

[37] Constantino JN, Gruber CP (2012). The Social Responsiveness Scale Manual, Second Edition (SRS-2).

[38] Kaufman AS, Kaufman NLKABC-II (2004). Kaufman assessment battery for children.

[39] Drozdick LW, Singer JK, Lichtenberger EO, Kaufman JC, Kaufman AS, Kaufman NL, Flanagan DP, McDonough EM (2018). The Kaufman Assessment battery for children—Second Edition and KABC-II normative update. Contemporary intellectual assessment: theories, tests, and issues.

[40] Khan S, Hashmi JA, Mamashli F, Michmizos K, Kitzbichler MG, Bharadwaj H (2018). Maturation trajectories of cortical resting-state networks depend on the mediating frequency band. Neuroimage.

[41] Orekhova EV, Rostovtseva EN, Manyukhina VO, Prokofiev AO, Obukhova TS, Nikolaeva AY (2020). Spatial suppression in visual motion perception is driven by inhibition: evidence from MEG gamma oscillations. Neuroimage.

[42] Stroganova TA, Komarov KS, Sysoeva OV, Goiaeva DE, Obukhova TS, Ovsiannikova TM (2020). Left hemispheric deficit in the sustained neuromagnetic response to periodic click trains in children with ASD. Mol Autism.

[43] Taulu S, Hari R (2009). Removal of magnetoencephalographic artifacts with temporal signal-space separation: demonstration with single-trial auditory-evoked responses. Hum Brain Mapp.

[44] Gramfort A, Luessi M, Larson E, Engemann DA, Strohmeier D, Brodbeck C (2014). MNE software for processing MEG and EEG data. Neuroimage.

[45] Uusitalo MA, Ilmoniemi RJ (1997). Signal-space projection method for separating MEG or EEG into components. Med Biol Eng Comput.

[46] Pascual-Marqui RD (2002). Standardized low-resolution brain electromagnetic tomography (sLORETA): technical details. Method Find Exp Clin.

[47] VanVeen BD, vanDrongelen W, Yuchtman M, Suzuki A (1997). Localization of brain electrical activity via linearly constrained minimum variance spatial filtering. IEEE Trans Bio-Med Eng.

[48] Hipp JF, Siegel M (2013). Dissociating neuronal gamma-band activity from cranial and ocular muscle activity in EEG. Front Hum Neurosci.

[49] Tait L, Ozkan A, Szul MJ, Zhang JX (2021). A systematic evaluation of source reconstruction of resting MEG of the human brain with a new high-resolution atlas: performance, precision, and parcellation. Hum Brain Mapp.

[50] Donoghue T, Haller M, Peterson EJ, Varma P, Sebastian P, Gao R (2020). Parameterizing neural power spectra into periodic and aperiodic components. Nat Neurosci.

[51] Andersen LM (2018). Group analysis in FieldTrip of time-frequency responses: a pipeline for reproducibility at every step of processing, going from individual sensor space representations to an across-group source space representation. Front Neurosci-Switz.

[52] Koo TK, Li MY (2016). A guideline of selecting and reporting intraclass correlation coefficients for reliability research. J Chiropr Med.

[53] Romei V, Brodbeck V, Michel C, Amedi A, Pascual-Leone A, Thut G (2008). Spontaneous fluctuations in posterior alpha-band EEG activity reflect variability in excitability of human visual areas. Cereb Cortex.

[54] Sauseng P, Klimesch W, Gerloff C, Hummel FC (2009). Spontaneous locally restricted EEG alpha activity determines cortical excitability in the motor cortex. Neuropsychologia.

[55] Zrenner C, Desideri D, Belardinelli P, Ziemann U (2018). Real-time EEG-defined excitability states determine efficacy of TMS-induced plasticity in human motor cortex. Brain Stimul.

[56] Muthukumaraswamy SD, Liley DTJ (2018). 1/f electrophysiological spectra in resting and drug-induced states can be explained by the dynamics of multiple oscillatory relaxation processes. Neuroimage.

[57] Dehghani N, Peyrache A, Telenczuk B, Quyen MLV, Halgren E, Cash SS (2016). Dynamic balance of excitation and inhibition in human and monkey neocortex. Sci Rep UK.

[58] Freund TF, Katona I (2007). Perisomatic inhibition. Neuron.

[59] Okun M, Lampl I (2008). Instantaneous correlation of excitation and inhibition during ongoing and sensory-evoked activities. Nat Neurosci.

[60] Harrington AJ, Raissi A, Rajkovich K, Berto S, Kumar J, Molinaro G (2016). MEF2C regulates cortical inhibitory and excitatory synapses and behaviors relevant to neurodevelopmental disorders. Elife.

[61] Satterstrom FK, Kosmicki JA, Wang JB, Breen MS, De Rubeis S, An JY (2020). Large-scale exome sequencing study implicates both developmental and functional changes in the neurobiology of autism. Cell.

[62] Decety J, Lamm C (2007). The role of the right temporoparietal junction in social interaction: how low-level computational processes contribute to meta-cognition. Neuroscientist.

[63] Saxe R, Kanwisher N (2003). People thinking about thinking people—the role of the temporo-parietal junction in "theory of mind". Neuroimage.

[64] Silani G, Lamm C, Ruff CC, Singer T (2013). Right supramarginal gyrus is crucial to overcome emotional egocentricity bias in social judgments. J Neurosci.

[65] Abu-Akel AM, Apperly IA, Wood SJ, Hansen PC (2017). Autism and psychosis expressions diametrically modulate the right temporoparietal junction. Soc Neurosci UK.

[66] Igelstrom KM, Webb TW, Graziano MSA (2017). Functional connectivity between the temporoparietal cortex and cerebellum in autism spectrum disorder. Cereb Cortex.

[67] Ramot M, Walsh C, Reimann GE, Martin A (2020). Distinct neural mechanisms of social orienting and mentalizing revealed by independent measures of neural and eye movement typicality. Commun Biol.

[68] Wang Q, Li HY, Li YD, Lv YT, Ma HB, Xiang AF (2021). Resting-state abnormalities in functional connectivity of the default mode network in autism spectrum disorder: a meta-analysis. Brain Imaging Behav.

[69] May KE, Kana RK (2020). Frontoparietal network in executive functioning in Autism Spectrum Disorder. Autism Res.

[70] Le Magueresse C, Monyer H (2013). GABAergic interneurons shape the functional maturation of the cortex. Neuron.

[71] Wang HX, Gao WJ (2009). Cell type-specific development of NMDA receptors in the interneurons of rat prefrontal cortex. Neuropsychopharmacology.

[72] Williamson JM, Lyons DA (2018). Myelin dynamics throughout life: an ever-changing landscape?. Front Cell Neurosci.

[73] Amlien IK, Fjell AM, Tamnes CK, Grydeland H, Krogsrud SK, Chaplin TA (2016). Organizing principles of human cortical development-thickness and area from 4 to 30 years: insights from comparative primate neuroanatomy. Cereb Cortex.

[74] Halgren M, Ulbert I, Bastuji H, Fabo D, Eross L, Rey M (2019). The generation and propagation of the human alpha rhythm. Proc Natl Acad Sci USA.

[75] Halgren M, Kang R, Voytek B, Ulbert I, Fabo D, Eross L, et al. The timescale and magnitude of 1/f aperiodic activity decrease with cortical depth in humans, macaques, and mice. 2021. 10.1101/2021.07.28.454235.

[76] Dehghani N, Bedard C, Cash SS, Halgren E, Destexhe A (2010). Comparative power spectral analysis of simultaneous elecroencephalographic and magnetoencephalographic recordings in humans suggests non-resistive extracellular media. J Comput Neurosci.

[77] Shirhatti V, Borthakur A, Ray S (2016). Effect of reference scheme on power and phase of the local field potential. Neural Comput.

[78] Houtman SJ, Lammertse HCA, van Berkel AA, Balagura G, Gardella E, Ramautar JR (2021). STXBP1 syndrome is characterized by inhibition-dominated dynamics of resting-state EEG. Front Physiol.

[79] Pathania A, Schreiber M, Miller MW, Euler MJ, Lohse KR (2021). Exploring the reliability and sensitivity of the EEG power spectrum as a biomarker. Int J Psychophysiol.

[80] Robinson PA, Rennie CJ, Wright JJ, Bahramali H, Gordon E, Rowe DL (2001). Prediction of electroencephalographic spectra from neurophysiology. Phys Rev E.

[81] Roche KJ, LeBlanc JJ, Levin AR, O'Leary HM, Baczewski LM, Nelson CA (2019). Electroencephalographic spectral power as a marker of cortical function and disease severity in girls with Rett syndrome. J Neurodev Disord.

[82] Tran TT, Rolle CE, Gazzaley A, Voytek B (2020). Linked sources of neural noise contribute to age-related cognitive decline. J Cogn Neurosci.

[83] Wilkinson CL, Nelson CA (2021). Increased aperiodic gamma power in young boys with Fragile X Syndrome is associated with better language ability. Mol Autism.

[84] Miller KJ, Sorensen LB, Ojemann JG, den Nijs M (2009). Power-law scaling in the brain surface electric potential. Plos Comput Biol.

[85] Wen HG, Liu ZM (2016). Separating fractal and oscillatory components in the power spectrum of neurophysiological signal. Brain Topogr.

[86] De Stefano P, Carboni M, Marquis R, Spinelli L, Seeck M, Vulliemoz S (2022). Increased delta power as a scalp marker of epileptic activity: a simultaneous scalp and intracranial electroencephalography study. Eur J Neurol.

[87] Newson JJ, Thiagarajan TC (2019). EEG frequency bands in psychiatric disorders: a review of resting state studies. Front Hum Neurosci.

[88] Ostrowski LM, Spencer ER, Bird LM, Thibert R, Komorowski RW, Kramer MA (2021). Delta power robustly predicts cognitive function in Angelman syndrome. Ann Clin Transl Neur.

[89] Muthukumaraswamy SD (2013). High-frequency brain activity and muscle artifacts in MEG/EEG: a review and recommendations. Front Hum Neurosci.

[90] Whithain EM, Pope KJ, Fitzgibbon SP, Lewis T, Clark CR, Loveless S (2007). Scalp electrical recording during paralysis: Quantitative evidence that EEG frequencies above 20 Hz are contaminated by EMG. Clin Neurophysiol.

[91] Whitham EM, Lewis T, Pope KJ, Fitzgibbon SP, Clark CR, Loveless S (2008). Thinking activates EMG in scalp electrical recordings. Clin Neurophysiol.

[92] Vandewouw MM, Dunkley BT, Lerch JP, Anagnostou E, Taylor MJ (2021). Characterizing inscapes and resting-state in MEG: effects in typical and atypical development. Neuroimage.

[93] Boto E, Holmes N, Leggett J, Roberts G, Shah V, Meyer SS (2018). Moving magnetoencephalography towards real-world applications with a wearable system. Nature.

[94] Iivanainen J, Stenroos M, Parkkonen L (2017). Measuring MEG closer to the brain: performance of on-scalp sensor arrays. Neuroimage.

[95] Schneiderman JF, Ruffieux S, Pfeiffer C, Riaz B, Supek S, Aine C (2019). On-ScalpMEG. Magnetoencephalography: from signals to dynamic cortical networks.

[96] Russell G, Mandy W, Elliott D, White R, Pittwood T, Ford T (2019). Selection bias on intellectual ability in autism research: a cross-sectional review and meta-analysis. Mol Autism.

