# Supplementary material for: Globally elevated excitation–inhibition ratio in children with autism spectrum disorder and below-average intelligence
Source: Mol Autism. 2022 May 12;13:20. doi: 10.1186/s13229-022-00498-2 (PMC9102291; doi:10.1186/s13229-022-00498-2)
Supplement: Supplementary file 1 — Additional file 1. Supplementary methods and results. [file 13229_2022_498_MOESM1_ESM.docx]

**Supplementary Information**

**Globally elevated excitation-inhibition ratio in children with autism spectrum disorder and below-average intelligence***

Viktoriya O. Manyukhina^1 ,2^ (MSc), Andrey O. Prokofyev^1^ (PhD), Ilia A. Galuta^1^ (MSc), Dzerassa E. Goiaeva^1^ (MSc), Tatiana S. Obukhova^1^ (MSc), Justin F. Schneiderman^3^ (PhD), Dmitry I. Altukhov^2^ (PhD), Tatiana A. Stroganova^1^ (PhD), Elena V. Orekhova^1^ (PhD)*

^1^ Center for Neurocognitive Research (MEG Center), Moscow State University of Psychology and Education, Moscow, Russian Federation

^2^ Department of Psychology, National Research University Higher School of Economics, Moscow, Russian Federation

^3^ MedTech West and the Institute of Neuroscience and Physiology, Sahlgrenska Academy, The University of Gothenburg, Gothenburg, Sweden

***** This study was supported by Russian Science Foundation (project # 22-25-00419).

**SUPPLEMENTARY METHODS**

**Participants: recruitment and exclusion details**

Fifty-eight typically developing (TD) boys and 63 boys with autism spectrum disorder (ASD), aged 6 to 15 years, were initially enrolled in this study. The TD children were recruited from local schools in Moscow and had no neurological or psychiatric disorders. The ASD children were recruited from rehabilitation centers affiliated with the Moscow University of Psychology and Education. Each participant in the ASD group had a diagnosis of ASD confirmed by an experienced psychiatrist according to the DSM-5 criteria and interviews with children’s parents or caregivers.

After visual inspection of MEG recordings, part of the children (9 and 13 TD, 14 and 25 ASD in the ‘eyes open’ (EO) and ’eyes closed’ (EC) conditions, respectively) were excluded from further MEG data analysis due to excessive muscle or other artifacts, or due to too large displacement of the head origin from the initial position (see below). The final number of participant datasets used for analysis was 49 TD and 49 ASD in the EO condition and 45 TD and 38 ASD in the EC condition. The number of participants of different age ranges is given in Table S1.

**Table S1 Number of participants of different age ranges**

|  | Age ranges | | |
| --- | --- | --- | --- |
|  | *6-9 years* | *9-12 years* | *12-15+ years* |
|  | Eyes open | | |
| TD | 14 | 21 | 14 |
| ASD, IQ>85 | 14 | 8 | 8 |
| ASD, IQ<85 | 6 | 9 | 4 |
|  | Eyes closed | | |
| TD | 12 | 19 | 14 |
| ASD, IQ>85 | 11 | 4 | 8 |
| ASD, IQ<85 | 6 | 7 | 2 |

**SUPPLEMENTARY RESULTS**

**Figure S1.**

**Fig. S1** Average power spectra under ‘eyes open’ **(A)** and ‘eyes closed’ **(B)** conditions in three groups of participants. The full-range (2-140 Hz) spectra represent the average of all gradiometer sensors. The inserts in the lower left corners show the average of all cortical sources (LCMV beamformer approach). Note that the signal was filtered in 30-140 Hz range before source localization.

**Contribution of myogenic artifacts to the group differences in spectral slope**

Muscle activity is the major source of high-frequency signals recorded by surface EEG^1^ and is a highly plausible source of MEG artifacts at frequencies >20 Hz^2^. Using a semiautomatic procedure for detection of myogenic artifacts (‘annotate_muscle_zscore’ function in MNE-python), we excluded epochs contaminated by *phasic* bursts of myogenic activity from our analyses. Still, *tonic* muscle activity may strongly contaminate the MEG signal recorded by planar gradiometers positioned in the vicinity of the cranial muscles. The power spectrum of the motor units’ activity is broad and is usually concentrated above 50 Hz^3^. Therefore, adding this activity to the MEG signal could potentially flatten the slope of the aperiodic component estimated in the 35-45 Hz frequency range.

Beamformers work as spatial filters that reconstruct activity from sources while suppressing interferences from all other sources, including those of muscle artifacts^4-6^. On the other hand, the least-squares minimum norm algorithms such as low resolution brain electromagnetic tomography (sLoreta)^7^ tend to overfit non-brain signals^8^ increasing the likelihood of misinterpreting myogenic activity as coming from brain sources. As beamformers reduce the myogenic contribution in the high frequency part of the spectrum, we expected that the spectral slopes estimated with them will be more negative than those estimated using sLoreta. The difference will furthermore be greatest in the regions closest to the sources of myogenic artifacts (i.e., at frontal, temporal, and occipital cortical areas). We also reasoned that if the group differences (TD, ASD_>85_, ASD_<85_) in the mean spectral slope of the aperiodic activity were driven by myogenic artifacts, they would be greater in sLoreta, as compared to beamformer, generated source estimates.

We therefore estimated the spectral slope of the signal localized with sLoreta in the same way as we did for signal localized using linearly constrained minimum variance (LCMV). For sLoreta localization, the MEG and empty room signals were band-passed in the 30-140 Hz range and the noise covariance matrix was derived from one minute of ‘MaxFiltered’ empty room data. The sLoreta inverse solution was estimated with the following parameters: semi-orthogonal orientation of the dipole source to the cortical surface (parameter loose set to 0.4), depth weighting parameter set to 0.8, and signal-to-noise ratio (SNR) set to 1. The inverse operator was applied separately to each one-second data epoch and the spectral analysis was performed at the source level in the same way as in case of LCMV beamformer.

As expected, the local spectral slope coefficients were more negative for the spectra estimated using the LCMV beamformer than for those estimated with sLoreta (Fig. S2A,B). Moreover, the differences in the slopes obtained with the two methods were greatest in the frontal, temporal, and occipital regions (Fig. S2C).


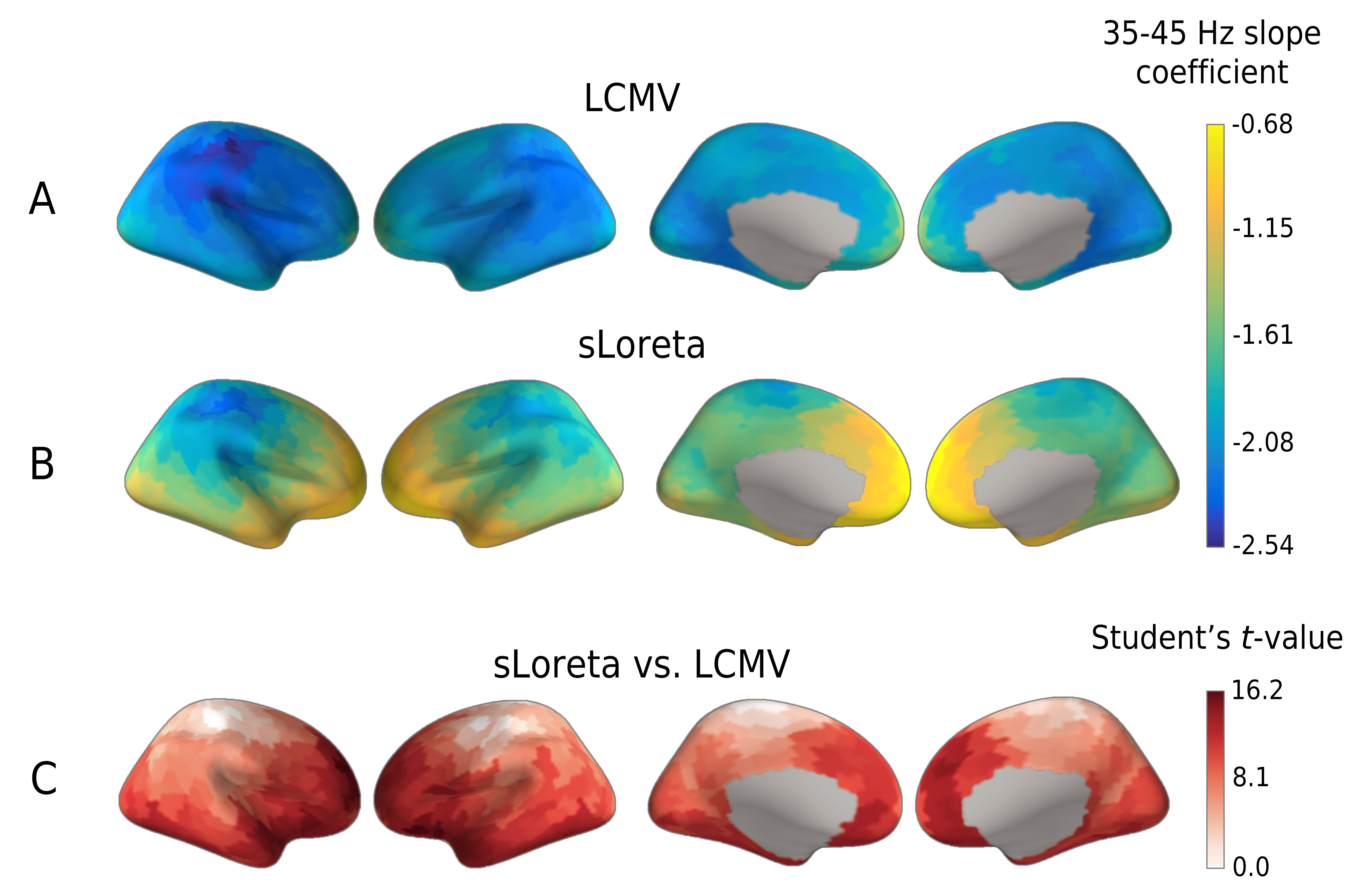


**Fig. S2** Inflated brain views of the spatial distribution of grand average (N=98) 35-45 Hz spectral slope coefficients of the aperiodic component estimated for the Eyes open condition using LCMV beamformer **(A)** and sLoreta **(B).** T-values **(C)** show the difference in the slopes estimated by the two methods (FDR corrected at p<0.05). Note that LCMV beamformer yields more negative slopes than sLoreta, but that the difference between the two methods is less significant (or not significant) in the regions most distant from the sources of myogenic artifacts (white spots at tops of brains). LCMV, linearly constrained minimum variance; sLoreta, standardized low resolution brain electromagnetic tomography; FDR, false discovery rate.

Next, we analyzed the group differences in the mean (over cortical labels) spectral slopes in the EO condition – for which the MEG data were available in all participants – using 1) beamformer and 2) sLoreta approaches to source estimation. In both cases the analysis of covariance (ANCOVA) with factors Group and Age revealed a significant effect of Group, but the statistical significance and size of this effect were higher in case of the LCMV beamformer (F_(2,94)_=8.4, p=0.00045, η^2^=0.15) than sLoreta (F_(2,94)_=3.6, p=0.032, η^2^=0.07). The differences in the mean spectral slope between ASD_<85_ and other experimental groups were greater for the LCMV beamformer (Fig. S3A) than sLoreta (Fig. S3B).

**Fig. S3** Mean spectral slope calculated in the three groups of participants using LCMV beamformer (A) and sLoreta **(B)** source estimation methods. Vertical bars denote 0.95 confidence intervals. Note that the group differences are greater in case of the LCMV beamformer, which is a method that is understood to produce source estimates that are less affected by myogenic activity, as compared to sLoreta. The group differences we report in the main text (derived from the LCMV estimates) are therefore unlikely to be explained by a group difference in the amount of myogenic activity. TD, typically developing children; ASD, autism spectrum disorder; ASD_>85_, children with ASD and Mental Processing Index above 85; ASD_<85_, children with ASD and Mental Processing Index below 85; LCMV, linearly constrained minimum variance; sLoreta, standardized low resolution brain electromagnetic tomography.

Inspection of Fig. S2 shows that, despite more negative slopes, the distribution of the slope coefficients estimated with LCMV beamformer is similar to that for the sLoreta: the LCMV-based slope coefficients are still less negative at the areas susceptible to myogenic contamination. Therefore, some residual contribution of myogenic artifacts to the high frequency activity, estimated with LCMV beamformers, could not be excluded. Nevertheless*,* the results presented in Fig. 4 of the main manuscript demonstrate that the majority of significant correlations of the local spectral slopes with MPI IQ are found in the regions relatively distant from the cranial muscles. The full list of the cortical labels where the significant correlations survived the FDR correction is presented in Table S2.

In general, this pattern of results suggests against the ‘myogenic nature’ of the observed group differences in the mean spectral slope.

**Table S2** The list of cortical labels^*^ where the FDR corrected partial Spearman correlations between the spectral slope and MPI IQ (controlling for age and sensitivity in the label) were significant in children with autism spectrum disorder

| Label name | Partial Spearman R | P-value |
| --- | --- | --- |
| *Right hemisphere* | | |
| bankssts_2-rh | -0.5 | 0.016 |
| bankssts_3-rh | -0.51 | 0.015 |
| inferiorparietal_1-rh | -0.46 | 0.022 |
| inferiorparietal_3-rh | -0.52 | 0.015 |
| inferiorparietal_4-rh | -0.59 | 0.005 |
| insula_1-rh | -0.37 | 0.05 |
| insula_7-rh | -0.39 | 0.044 |
| lateralorbitofrontal_3-rh | -0.39 | 0.044 |
| middletemporal_4-rh | -0.39 | 0.044 |
| paracentral_4-rh | -0.51 | 0.015 |
| paracentral_5-rh | -0.44 | 0.027 |
| paracentral_6-rh | -0.43 | 0.028 |
| parsopercularis_4-rh | -0.38 | 0.046 |
| parsorbitalis_1-rh | -0.43 | 0.028 |
| parstriangularis_1-rh | -0.42 | 0.029 |
| parstriangularis_3-rh | -0.38 | 0.049 |
| postcentral_3-rh | -0.37 | 0.049 |
| postcentral_4-rh | -0.45 | 0.027 |
| postcentral_7-rh | -0.42 | 0.029 |
| postcentral_8-rh | -0.44 | 0.027 |
| posteriorcingulate_2-rh | -0.39 | 0.045 |
| posteriorcingulate_4-rh | -0.52 | 0.015 |
| precentral_10-rh | -0.43 | 0.028 |
| precentral_11-rh | -0.47 | 0.019 |
| precentral_12-rh | -0.39 | 0.045 |
| precentral_6-rh | -0.4 | 0.041 |
| precentral_7-rh | -0.47 | 0.022 |
| precentral_9-rh | -0.39 | 0.044 |
| precuneus_10-rh | -0.37 | 0.049 |
| rostralanteriorcingulate_2-rh | -0.4 | 0.043 |
| rostralmiddlefrontal_10-rh | -0.45 | 0.026 |
| rostralmiddlefrontal_3-rh | -0.39 | 0.045 |
| rostralmiddlefrontal_5-rh | -0.39 | 0.044 |
| rostralmiddlefrontal_6-rh | -0.39 | 0.044 |
| rostralmiddlefrontal_8-rh | -0.4 | 0.043 |
| superiorfrontal_1-rh | -0.39 | 0.044 |
| superiorfrontal_2-rh | -0.49 | 0.019 |
| superiorfrontal_3-rh | -0.45 | 0.027 |
| superiortemporal_1-rh | -0.38 | 0.049 |
| superiortemporal_2-rh | -0.42 | 0.031 |
| supramarginal_1-rh | -0.45 | 0.026 |
| supramarginal_2-rh | -0.44 | 0.027 |
| supramarginal_3-rh | -0.47 | 0.019 |
| supramarginal_4-rh | -0.44 | 0.027 |
| supramarginal_5-rh | -0.42 | 0.03 |
| supramarginal_6-rh | -0.43 | 0.028 |
| supramarginal_7-rh | -0.44 | 0.027 |
| supramarginal_8-rh | -0.54 | 0.015 |
| supramarginal_9-rh | -0.47 | 0.019 |
| *Left hemisphere* | | |
| cuneus_1-lh | -0.38 | 0.048 |
| cuneus_2-lh | -0.4 | 0.043 |
| inferiorparietal_2-lh | -0.44 | 0.027 |
| insula_1-lh | -0.42 | 0.029 |
| insula_6-lh | -0.4 | 0.041 |
| lateraloccipital_3-lh | -0.51 | 0.015 |
| parsopercularis_1-lh | -0.38 | 0.046 |
| parstriangularis_2-lh | -0.48 | 0.019 |
| parstriangularis_3-lh | -0.4 | 0.043 |
| pericalcarine_1-lh | -0.4 | 0.041 |
| pericalcarine_2-lh | -0.43 | 0.028 |
| postcentral_11-lh | -0.44 | 0.027 |
| postcentral_12-lh | -0.38 | 0.048 |
| postcentral_13-lh | -0.41 | 0.036 |
| postcentral_14-lh | -0.38 | 0.049 |
| precentral_11-lh | -0.4 | 0.043 |
| precentral_12-lh | -0.38 | 0.049 |
| precentral_8-lh | -0.42 | 0.031 |
| rostralanteriorcingulate_2-lh | -0.44 | 0.028 |
| rostralmiddlefrontal_1-lh | -0.4 | 0.041 |
| rostralmiddlefrontal_10-lh | -0.42 | 0.029 |
| rostralmiddlefrontal_4-lh | -0.48 | 0.019 |
| rostralmiddlefrontal_5-lh | -0.44 | 0.027 |
| rostralmiddlefrontal_6-lh | -0.43 | 0.028 |
| rostralmiddlefrontal_7-lh | -0.43 | 0.028 |
| rostralmiddlefrontal_8-lh | -0.49 | 0.019 |
| rostralmiddlefrontal_9-lh | -0.48 | 0.019 |
| superiorfrontal_2-lh | -0.45 | 0.026 |
| superiorfrontal_3-lh | -0.49 | 0.019 |
| superiorfrontal_4-lh | -0.42 | 0.029 |
| superiorparietal_10-lh | -0.39 | 0.045 |
| superiortemporal_11-lh | -0.38 | 0.049 |
| superiortemporal_2-lh | -0.38 | 0.049 |
| superiortemporal_4-lh | -0.37 | 0.05 |
| supramarginal_1-lh | -0.46 | 0.022 |
| supramarginal_2-lh | -0.53 | 0.015 |
| supramarginal_3-lh | -0.46 | 0.022 |
| transversetemporal_2-lh | -0.43 | 0.028 |

^*^ Parcellation into 448 cortical similar-size labels was performed as described by Khan et al^9^.

MPI, Mental Processing Index; FDR, false discovery rate.

**Effect of background noise and motion correction on the spectral slope and high-frequency power**

There are two main sources of non-biological (instrumental) noise that may confound spectral power and slope estimation in the high frequency range.

One is the background (‘empty room’) noise. A high power level of spectrally flat (‘white’) background noise (Fig. 1 in the main manuscript) may result in flattening of the spectral slope measured in the high frequency range. To estimate the background noise, we calculated the mean over all gradiometers power of the ‘empty room’ signal in the 35-45 Hz range for each subject’s dataset.

The other is the noise introduced by the head motion correction procedure. This noise is proportional to the transformation distance^10^. To estimate the noise associated with the motion correction procedure, for each subject we calculated the mean transformation distance (displacement of the ‘head origin’ from the initial position) during the respective condition.

Table S3 shows the correlation of the instrumental noise with the averaged over all cortical labels 35-45 Hz power estimated with sLoreta and the averaged over all cortical labels spectral slope coefficients estimated with the two source localization methods (LCMV beamformer and sLoreta). The correlations were calculated for the pooled sample of participants (N=98) in the EO condition, for which data from all participants were available.

**Table S3** Spearman correlations of the 35-45 Hz mean spectral power and slope (eyes open condition) with instrumental noise in the pooled group of participants

|  | Mean  empty room noise | Mean  transformation distance |
| --- | --- | --- |
| *Mean 35-45 Hz power* | | |
| *sLoreta* | R_(98)_=0.16, p=0.12 | **R_(98)_=0.23, p=0.02** |
| *Mean slope coefficient* | | |
| *sLoreta* | **R_(98)_=0.33, p=0.0011** | R_(98)_=0.07, p=0.52 |
| *LCMV beamformer* | **R_(98)_=0.24, p=0.016** | R_(98)_=-0.02, p=0.85 |

LCMV, linearly constrained minimum variance; sLoreta, standardized low resolution brain electromagnetic tomography.

As expected, a higher magnitude of the empty room noise correlated with a flatter spectral slope, estimated with either beamformer or sLoreta approaches. The transformation distance, however, did not affect the grand average spectral slope in a systematic way. The correlations of the transformation distance with spectral slopes estimated in the 448 individual labels were also all not significant (only 1 of 448 correlations for the ‘sLoreta-estimated’ slopes and 5 of 448 correlations for the ‘beamformer-estimated’ slopes displayed 0.01<p’s<0.05, uncorrected for multiple comparisons).

The grand average 35-45 Hz spectral power correlated positively with the mean transformation distance. Although the correlation was low (R_(98)_=0.23, p=0.02), inspection of the correlations in the individual labels revealed that they were highest (R~0.5) in the regions most distant from the cranial muscles (caudal midfrontal, paracentral, precentral, postcentral), i.e., in the areas where instrumental noise might be the main source of MEG signal contamination.

Considering possible contribution of instrumental noise and myogenic artifacts to the absolute high-frequency spectral power, we suggested that analysis of the group differences in the absolute high-frequency power or 1/f intercept might produce unreliable results. We have therefore chosen not to analyze these parameters.

To ensure that the instrumental noise does not significantly contribute to the observed correlation between the spectral slope and IQ in children with ASD (Fig. 3C in the main manuscript), we used the mean empty room noise power in 35-45 Hz range and transformation distance as nuisance variables while calculating partial Spearman correlations between mean spectral slope and IQ in children with ASD. After accounting for these confounding variables, the correlations between the mean spectral slope and IQ remained significant (EO: N=49, R_partial_=-0.44, p=0.002; EC: N=38, R_partial_=-0.38, p=0.02).

# Estimation of the spectral slope with FOOOF – ‘Fitting Oscillations & One Over F’

To estimate the slope of aperiodic component of the spectra in the 2-45 Hz range with ‘fitting oscillations & one over f’ (FOOOF)^11^ (hereinafter referred to as the ‘2-45 Hz spectral slope’), we first filtered the raw signal between 1-47 Hz. The other steps of the source localization and spectral estimation were the same as for the 35-45 Hz spectral slope. For spectrum parameterization in the 2-45 Hz range we used the FOOOF Python package (<https://pypi.python.org/pypi/fooof/>; v.1.0.0). We extracted aperiodic components from each of the 448 labels’ power spectrum with the option ‘fixed’ (no knee); other parameters were set to default. By multiplying by -1, the scaling exponent provided by FOOOF was converted into the linear slope coefficient on a logarithmic scale. We then calculated the mean (over cortical labels) 2-45 Hz spectral slope in the same way as we did for the slope in the 35-45 Hz range.

Results of ANCOVA with factors Group (TD, ASD_>85_, ASD_<85_), Condition (EO, EC), and Age are shown in Table S4. Unlike the spectral slope directly measured in the 35-45 Hz range, the slope estimated in the broad frequency range (2-45 Hz) using FOOOF did not reveal group differences (Effect of Group: F_(2,80)_=0.72, p=0.49, η^2^=0.018; Table S4).

**Table S4** Results of general linear model analysis for the 2-45 Hz spectral slope estimated with FOOOF: effects of Group (TD, ASD_>85_, ASD_<85_), Condition (eyes closed, eyes open), and Age

| rmANOVA effect | F_(df)_, p, η^2^ |
| --- | --- |
| Group | F_(2,79)_=0.69, p=0.50, η^2^ =0.017 |
| Age | F_(1,79)_=29.67, p=1e-6, η^2^ =0.27 |
| Condition | F_(1,79)_=16.42, p=0.0001, η^2^=0.17 |
| Condition x Age | F_(1,79)_=1.51, p=0.22, η^2^=0.019 |
| Condition x Group | F_(2,79)_=0.56, p=0.57, η^2^=0.014 |

TD, typically developing children; ASD, autism spectrum disorder; ASD_>85_, children with ASD and Mental Processing Index above 85; ASD_<85_, children with ASD and Mental Processing Index below 85; rmANOVA, repeated measures analysis of variance.

Although the difference in results obtained using the two methods of the slope estimation requires further investigation, we believe that, in our study, the spectral slope might not be optimally estimated in the 2-45 Hz range. *Firstly*, a poorly detectable spectral ‘knees’ might present in the data^12-14^ and vary among participants and cortical locations. Since detection of a ‘knee’ in the noisy data and with our 1 Hz frequency resolution was problematic, we used ‘no knee’ approach, which could distort estimation of aperiodic activity. *Secondly*, while the FOOOF relies on the assumption that all oscillation peaks are lying within the fitting range (i.e., in our case 2-45 Hz), this might not always be the case in our participants, especially in those with ASD, in whom power of delta-range oscillations could be increased depending on the severity of their condition^15-17^. This might seriously compromise the results, as discussed in a recent paper by Gerster et al^18^. When fitting and removing the oscillation peaks seems unfeasible, Gerster et al. recommend estimating the slope of aperiodic component (1/f exponent) at high frequencies. *Thirdly,* the source estimation of the low-amplitude high-frequency activity with LCMV beamformers may be imprecise when estimates are based on relatively broadband (2-45 Hz) data. This because beamformer weights that are computed from broadband data are inherently biased towards resolving higher-amplitude low-frequency brain activity^19^.

**REFERENCES**

1. Whitham, E. M., et al., Thinking activates EMG in scalp electrical recordings. *Clin Neurophysiol* **119**, 1166-1175 (2008).

2. Muthukumaraswamy, S. D., High-frequency brain activity and muscle artifacts in MEG/EEG: a review and recommendations. *Front Hum Neurosci* **7**, (2013).

3. Farina, D.; Merletti, R.; Enoka, R. M., The extraction of neural strategies from the surface EMG. *J Appl Physiol* **96**, 1486-1495 (2004).

4. Hillebrand, A.; Barnes, G. R., Beamformer analysis of MEG data. *Int Rev Neurobiol* **68**, 149-+ (2005).

5. Hipp, J. F.; Siegel, M., Dissociating neuronal gamma-band activity from cranial and ocular muscle activity in EEG. *Front Hum Neurosci* **7**, (2013).

6. VanVeen, B. D.; vanDrongelen, W.; Yuchtman, M.; Suzuki, A., Localization of brain electrical activity via linearly constrained minimum variance spatial filtering. *Ieee T Bio-Med Eng* **44**, 867-880 (1997).

7. Pascual-Marqui, R. D., Standardized low-resolution brain electromagnetic tomography (sLORETA): Technical details. *Method Find Exp Clin* **24**, 5-12 (2002).

8. Tait, L.; Ozkan, A.; Szul, M. J.; Zhang, J. X., A systematic evaluation of source reconstruction of resting MEG of the human brain with a new high-resolution atlas: Performance, precision, and parcellation. *Hum Brain Mapp* **42**, 4685-4707 (2021).

9. Khan, S., et al., Maturation trajectories of cortical resting-state networks depend on the mediating frequency band. *Neuroimage* **174**, 57-68 (2018).

10. Neuromag, E. *MaxFilter User's Guide*, 2.2; 2010.

11. Donoghue, T., et al., Parameterizing neural power spectra into periodic and aperiodic components. *Nat Neurosci* **23**, 1655-U288 (2020).

12. Colombo, M. A., et al., The spectral exponent of the resting EEG indexes the presence of consciousness during unresponsiveness induced by propofol, xenon, and ketamine. *Neuroimage* **189**, 631-644 (2019).

13. He, B. Y. J.; Zempel, J. M.; Snyder, A. Z.; Raichle, M. E., The Temporal Structures and Functional Significance of Scale-free Brain Activity. *Neuron* **66**, 353-369 (2010).

14. Robinson, P. A., et al., Prediction of electroencephalographic spectra from neurophysiology. *Phys Rev E* **63**, (2001).

15. Capal, J. K., et al., EEG endophenotypes in autism spectrum disorder. *Epilepsy Behav* **88**, 341-348 (2018).

16. Newson, J. J.; Thiagarajan, T. C., EEG Frequency Bands in Psychiatric Disorders: A Review of Resting State Studies. *Front Hum Neurosci* **12**, (2019).

17. Wang, J., et al., Resting state EEG abnormalities in autism spectrum disorders. *J Neurodev Disord* **5**, (2013).

18. Gerster, M., et al., Separating neural oscillations from aperiodic 1/f activity: challenges and recommendations. *Preprint at* <https://doi.org/10.1101/2021.10.15.464483> (2021).

19. Dalal, S. S., et al., Five-dimensional neuroimaging: Localization of the time-frequency dynamics of cortical activity. *Neuroimage* **40**, 1686-1700 (2008).
